# Endocranial Anatomy of the Charadriiformes: Sensory System Variation and the Evolution of Wing-Propelled Diving

**DOI:** 10.1371/journal.pone.0049584

**Published:** 2012-11-27

**Authors:** N. Adam Smith, Julia A. Clarke

**Affiliations:** Department of Geological Sciences, Jackson School of Geosciences, University of Texas at Austin, Austin, Texas, United States of America; University of Lethbridge, Canada

## Abstract

Just as skeletal characteristics provide clues regarding behavior of extinct vertebrates, phylogenetically-informed evaluation of endocranial morphology facilitates comparisons among extinct taxa and extant taxa with known behavioral characteristics. Previous research has established that endocranial morphology varies across Aves; however, variation of those systems among closely related species remains largely unexplored. The Charadriiformes (shorebirds and allies) are an ecologically diverse clade with a comparatively rich fossil record, and therefore, are well suited for investigating interspecies variation, and potential links between endocranial morphology, phylogeny, ecology and other life history attributes. Endocranial endocasts were rendered from high resolution X-ray computed tomography data for 17 charadriiforms (15 extant and two flightless extinct species). Evaluation of endocranial character state changes on a phylogeny for Charadriiformes resulted in identification of characters that vary in taxa with distinct feeding and locomotor ecologies. In comparison with all other charadriiforms, stem and crown clade wing-propelled diving Pan-Alcidae displayed compressed semicircular canals, and indistinct occipital sinuses and cerebellar fissures. Flightless wing-propelled divers have relatively smaller brains for their body mass and smaller optic lobes than volant pan-alcids. Observed differences between volant and flightless wing-propelled sister taxa are striking given that flightless pan-alcids continue to rely on the flight stroke for underwater propulsion. Additionally, the brain of the Black Skimmer *Rynchops niger*, a taxon with a unique feeding ecology that involves continuous forward aerial motion and touch-based prey detection used both at day and night, is discovered to be unlike that of any other sampled charadriiform in having an extremely large wulst as well as a small optic lobe and distinct occipital sinus. Notably, the differences between the Black Skimmer and other charadriiforms are more pronounced than between wing-propelled divers and other charadriiforms. Finally, aspects of endosseous labyrinth morphology are remarkably similar between divers and non-divers, and may deserve further evaluation.

## Introduction

Although variation in brain and inner ear morphology across Aves has been documented [1]–[4], variation within major avian sub-clades has received comparatively little attention. Previous studies of avian endocranial anatomy have largely been limited to evaluations of single species or relatively small quantities of taxa that limited phylogenetically contextualized inferences about the distribution or potential homoplasy of morphological characters observed [5]–[9]. Furthermore, rather than assessing broad-scale morphological trends among taxa, studies with dense taxonomic sampling have often focused on particular endocranial features such as the olfactory lobe, the wulst (i.e., sagittal eminence), or relative size of different brain regions [10]–[14]. Recent studies of moa (Dinornithiformes), Haast's Eagle *Harpagornis moorei* (Accipitridae) and penguins (Pan-Sphenisciformes) [15]–[17] provide some of the first examples of endocranial studies that sampled multiple closely related species and examined in detail, the morphological differences among the endocranial anatomy of those taxa.

**Table 1:** Foraging behavior, nesting dispersion, and developmental strategies of sampled charadriiform taxa.

Taxon	Species Sampled	English Name	Specimen Number	Foraging Ethology	Nesting Ethology	Develop- ment	Reference
Charadriiformes	*Aethia cristatella*	Crested Auklet	NCSM 17749	WPD	C	P	+
	*Fratercula corniculata*	Horned Puffin	NCSM 18083	WPD	C	P	+
	*Fratercula cirrhata*	Tufted Puffin	EB 01490	WPD	C	P	[43]
	*Fratercula arctica*	Atlantic Puffin	Unknown	WPD	C	P	[44]
	*Alle alle*	Dovekie	NCSM 22391	WPD	C	P	+
	*Uria aalge*	Common Murre	NCSM 18117	WPD	C	SP	+
	*Alca torda*	Razorbill Auk	NCSM 20058	WPD	C	SP	+
	*Pinguinus impennis* †	Great Auk	USNM 623465	FWPD	C	*	+
	*Cepphus columba*	Pigeon Guillemot	NCSM 18095	WPD	C	P	+
	*Brachyramphus marmoratus*	Marbled Murrelet	NCSM 18149	WPD	S	P	+
	*Synthliboramphus antiquus*	Ancient Murrelet	NCSM 18090	WPD	C	SP	+
	Mancallinae †	Lucas Auk	SDSNH 25373	FWPD	*	*	+
	*Stercorarius longicaudus*	Long-tailed Skua	NCSM 10269	G	C	P	+
	*Rynchops niger*	Black Skimmer	NCSM 19603	ASS	C	P	+
	*Larus argentatus*	Herring Gull	NCSM 8624	G	C	SP	+ [44]
	*Larus crassirostris*	Black-tailed Gull	EB 01171	G	C	SP	[43]
	*Rissa tridactyla*	Black-legged Kittiwake	NCSM 18123	APD/ASS	C	SP	+
	*Sterna anaethetus*	Bridled Tern	NCSM 17085	APD/ASS	C	SP	+
	*Stiltia isabella*	Australian Pratincole	AMNH 9599	TGF	C	P	+
	*Rostratula benghalensis*	Greater Painted Snipe	EB 03636	TGF	S	P	[43]
	*Tringa stagnatilis*	Marsh Sandpiper	EB 02921	TGF	C	SP	[43]
	*Scolopax rusticola*	Eurasian Woodcock	Unknown	TGF	S	SP	[44]
	*Calidris alpina*	Dunlin	Unknown	TGF	S	P	[50]
	*Vanellus miles*	Masked Lapwing	Unknown	TGF	S	SP	[50]
	*Vanellus vanellus*	Northern Lapwing	Unknown	TGF	S	SP	[44]
	*Pluvialis squatarola*	Grey Plover	Unknown	TGF	S	SP	[44]
	*Pluvialis apricaria*	Eurasian Golden Plover	Unknown	TGF	S	SP	[44]
	*Pluvianus aegyptius*	Egyptian Plover	Unknown	TGF	S	P	[44]
	*Burhinus oedicnemus*	Eurasian Stone Curlew	Unknown	TGF	S	P	[44]
	*Charadrius vociferous*	Killdeer	NCSM 18305	TAF/TGF	S	P	+
	*Halcyornis toliapicus* †	–	NHMUK A130	*	*	*	[9]

Extinct species are denoted by a "†". *Ethological and developmental attributes of Mancallinae and *Halcyornis toliapicus* are largely unknown because these extinct taxa are known only from fossils. Taxa CT scanned for this study are denoted by a "+". Abbreviations: APD, aerial plunge diver; ASS, aerial surface skimmer; C, colonial; G, generalist; FWPD, flightless wing-propelled diver; P, precocial; S, solitary; SP, semi-precocial; TAF, terrestrial aerial forager; TGF, terrestrial ground-forager; WPD, wing-propelled diver.

Some evidence suggests that overall brain shape may be fairly conserved among clades and, therefore, it has been proposed that endocranial morphology may be phylogenetically informative; however, variation in endocranial morphology and the shape of features such as semicircular canals has been both linked to locomotor style and other ecological attributes as well as phylogeny [1], [18], [19]. Furthermore, the behavior, cognitive ability, and potential adaptability of extant birds has been linked with the relative size and gross morphology of different regions of the brain (e.g., [20]–[23]). For example, it has been suggested that differences in the size of the telencephalon in parrots and pigeons may been linked with differences in the cognitive ability of those taxa [22]. The brains of relatively distantly related birds that share similar ecologies often share morphological similarities (i.e., cerebrotypes, sensu [1]). However, whether closely related species of birds that display a range of ecological attributes also display a range of different brain morphologies has not been examined in many clades (i.e., just how conservative is brain morphology within clades). The study of cerebrotypes (i.e., brain categories based on the relative size of different brain regions) by Iwaniuk and Hurd (2005) used multivariate statistics and a cluster analysis to evaluate potential relationships between cerebrotype, ecology, and phylogeny in 67 species of birds. Those authors recovered a strong relationship between cerebrotype and a set of evaluated ecological attributes (e.g., locomotor style, mode of predation, cognitive ability) but also a less strongly supported relationship among cerebrotypes within major clades of birds (e.g., most galliforms have similar brain morphology). Although only five charadriiforms were included in that study, Iwaniuk and Hurd (2005) found that shorebirds and their allies displayed more brain variation than any other clade in their sample.

**Table 2:** Foraging behavior, nesting dispersion, and developmental strategies of sampled outgroup taxa to Charadriiformes.

Strigiformes	*Bubo virginianus*	Great Horned Owl	OUVC 10220	AP	S	A	[2]
	*Tyto alba*	Barn Owl	Unknown	AP	S	A	[12], [13]
Passeriformes	*Corvus moneduloides*	New Caledonian Crow	Unknown	G	C	A	[23]
	*Acridotheres tristis*	Common Myna	EB 03404	TGF	S	A	[43]
Sphenisciformes	*Paraptenodytes antarcticus* †	–	AMNH 3338	FWPD	C	*	[17], [45]
	*Pygocelis antarctica*	Chinstrap Penguin	AMNH 26121	FWPD	C	*	[17], [45]
Gaviiformes	*Gavia immer*	Common Loon	TCWC 13300	FPD	S	P	[17], [45]
Pelecaniformes	*Phalacrocorax carbo*	Great Cormorant	EB 07227	FPD	C	A	[43]
	*Prophaethon shrubsolei* †	–	NHMUK 44096	*	*	*	[8]
Pelagornithidae	*Odontopteryx toliapica* †	Pseudotooth	NHMUK A683	ASS	*	*	[8]
Columbiformes	*Columbia livia*	Rock Dove	NSM PO	TGF	S	A	[13], [43]
Galloanserae	*Anas platyrynchos*	Mallard	EPSM-AV 1209	G	S	P	[18], [43]
	*Chauna chauvaria*	Northern Screamer	KU 81969	TGF	S	P	[18]
	*Gallus gallus*	Red Junglefowl	Unknown	TGF	S	P	[43]
	*Phasianus versicolor*	Green Pheasant	Unknown	TGF	S	P	[43]
Palaeognathae	*Struthio camelus*	Ostrich	Unknown	TGF	S	P	[13], [43]
	*Rhea americana*	Rhea	MLP 824	TGF	S	P	[7]
	*Dinornis novazealandiae* †	North Island Giant Moa	LB 7082	TGF	S	*	[13]
	*Dinornis giganteus* †	South Island Giant Moa	AMNH FR1000001	TGF	S	*	[18]

Extinct species are denoted by a “†”. *Ethological and developmental attributes of *Prophaethon shrubsolei* and *Odontopteryx toliapica* are largely unknown because these extinct taxa are known only from fossils; however, pseudotoothed birds such as *Odontopteryx* have been interpreted as aerial surface skimmers [84]. Abbreviations: A, altricial; AP  =  aerial predator; ASS, aerial surface skimmer; C, colonial; FPD, foot-propelled diver; G, generalist; FWPD, flightless wing-propelled diver; P, precocial; S, solitary; SP, semi-precocial; TAF, terrestrial aerial forager; TGF, terrestrial ground-forager.

Morphological and functional variation has also been identified in the endosseous labyrinths of birds [17], [19], [24]. The tissues associated with the endosseous labyrinth serve multiple functions in vertebrates including, but not limited to, hearing and balance [24]. A variety of modifications to the outer ear of aquatic birds keep water out of the external auditory canal, and provide evidence that hearing in aquatic birds is adapted for the terrestrial or aerial environment rather than the aquatic environment [25]. Hearing plays little if any role in underwater predation by birds (i.e., underwater feeding of aquatic birds is visually mediated); however, aquatic birds reproduce on land, and vocally communicate at the surface of the water and on land [25]. Thus, because characteristics such as cochlea duct length have been correlated with complexity of vocalization [3], which has in turn been correlated with sociality [3], differences in the inner ear of colony and solitary nesters were explored.

**Figure 1:**
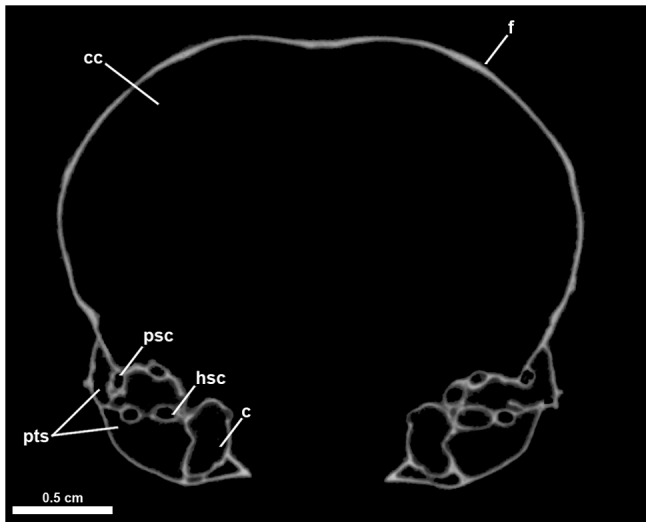
Representative coronal slice through the skull of the Marbled Murrelet *Brachyramphus marmoratus* (NCSM 18149).

Charadriiformes (sandpipers, plovers, gulls, terns, auks and allies) are an ecologically and behaviorally diverse clade [26] with well supported, and largely congruent, current hypotheses of relationships based on both molecular sequence and combined morphological-molecular datasets [27]–[29]. Recent hypotheses of systematic relationships among extant and extinct charadriiforms [27], [28], [30], [31] provide a phylogenetic framework on which inferences regarding potential correlation of endocranial morphology with ecological and ethological attributes may be evaluated. Relatively dense taxonomic sampling of a single clade that displays a variety of locomotor styles as well as specialist and generalist feeding ecologies that range from dominantly terrestrial foraging to aerial and pelagic strategies (i.e., 31 species of extant and extinct charadriiforms and 19 outgroup taxa; Tables 1, 2) allows for inferences regarding the evolution of charadriiform endocranial morphology and potential links with sensory system development to be explored in a phylogenetic context. Specifically, these data allow exploration of the transition to wing-propelled diving in Charadriiformes, an independent transition from that seen in penguins, which fly underwater in different ways than pan-alcids [32], [33]. Additionally, because the endocranial morphology of flightless pan-alcids has not been previously documented, the discovery of endocranial characteristics shared with penguins may provide clues to broader trends in the evolution of flightless wing-propelled divers across Aves.

**Table 3:** Endocranial volume, endosseous labyrinth volume, body mass and encephalization quotients (EQ) of sampled charadriiform taxa.

Taxon	Endocranial Volume (cm^3^)	Endosseous Labyrinth Volume (cm^3^)	Body Mass (g)	EQ
*Aethia cristatella*	2.3532	0.0254	310.0	0.660
*Fratercula corniculata*	4.9045	0.0287	647.0	0.906
*Alca torda*	5.2023	0.0314	485.4	1.131
*Pinguinus impennis*	11.018	0.0633	5000.0*	0.637
*Uria aalge*	4.7528	0.0309	776.0	0.792
*Alle alle*	1.8936	0.0139	175.0*	0.735
*Brachyramphus marmoratus*	2.1452	0.0148	220.0*	0.731
*Synthliboramphus antiquus*	2.0985	0.0149	207.0*	0.741
*Cepphus columba*	3.6700	0.0227	328.0	0.998
Mancallinae sp.	4.4949	0.0365	1650.0*	0.488
*Stercorarius longicaudus*	2.7524	0.0268	337.9	0.736
*Larus argentatus*	6.1264	0.0434	1147.0*	0.818
*Rissa tridactyla*	3.8868	0.0198	189.0	1.444
*Sterna anaethetus*	2.1377	0.0149	125.0	1.005
*Rynchops niger*	2.4529	0.0198	254.0*	0.708
*Stiltia isabella*	1.0115	0.0131	65.5*	0.686
*Charadrius vociferus*	1.2641	0.0173	107.9	0.646

Inner ear endosseous labyrinth volume was calculated by averaging computed values for the right and left labyrinths. Body mass values denoted by an ‘*’ are from previously published sources [26], [38] owing to lack of data for sampled skeletonized specimens. Based on comparisons with other known Mancallinae skulls (e.g., SDSNH 25236, SDSNH 68312; [28]), the size of the Mancallinae sp. skull (SDSNH 23753) sampled represents a small individual likely corresponding to the size of *Mancalla vegrandis*. The size of *Mancalla vegrandis* correlates with values attributed to ‘*Mancalla milleri*’ in the morphometric study of Livezey [28], [34], and thus, the mass estimated for ‘*Mancalla milleri*’ by Livezey (1988) is used here.

Here we document endocranial anatomy across Charadriiformes from rendered computed tomography (CT) data as well as considering these data in the context of variables such as body size and aspects of ecological habitus. Twenty-eight discrete morphological characters were used to describe observed morphological variation among sampled taxa and to evaluate the optimization of these features on a phylogeny of Charadriiformes [28]. With this work we address the following four broad questions: (**1**) How does the within-clade endocranial morphological variation among charadriiforms compare to the morphological differences documented for other avian clades; and, is charadriiform endocranial morphology relatively conserved despite ecological and ethological differences? Moreover, are there endocranial apomorphies of Charadriiformes relative to outgroup taxa that might be used to identify fossil charadriiform taxa? (**2**) Are there endocranial apomorphies that characterize clades within Charadriiformes? For example, are there differences in the endocranial morphology of wing-propelled diving Pan-Alcidae relative to volant charadriiform taxa given that extensive osteological modifications in pan-alcids have been previously documented [28], [31], [34]? (3) Is the relative volume of the charadriiform endocranial cavity correlated with variables such as endosseous labyrinth volume, body mass, phylogeny, flightlessness, developmental strategies and dive depth. For example, are the endocranial volumes of flightless charadriiforms relatively different from those of other closely related taxa? (**4**) Are the relative proportions and morphology of specific regions of the charadriiform brain and inner ear (e.g., optic lobe) correlated with specializations for different sensory modalities (i.e., associated with differences in locomotion, olfaction, proprioception, or feeding ecology) as has been shown in other clades (e.g., locomotion and olfaction in waterfowl, [10], [14], [24])?

**Figure 2:**
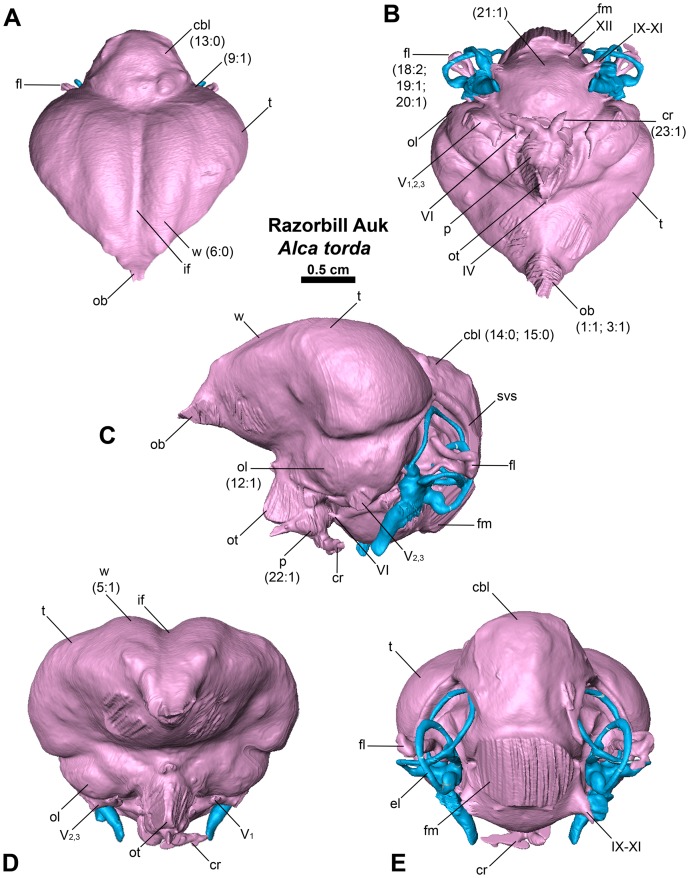
Endocast of the Razorbill Auk *Alca torda* in dorsal (A), ventral (B), lateral (C), anterior (D), and posterior (E) views. Numbers in parentheses represent character states from Appendix S1.

**Figure 3:**
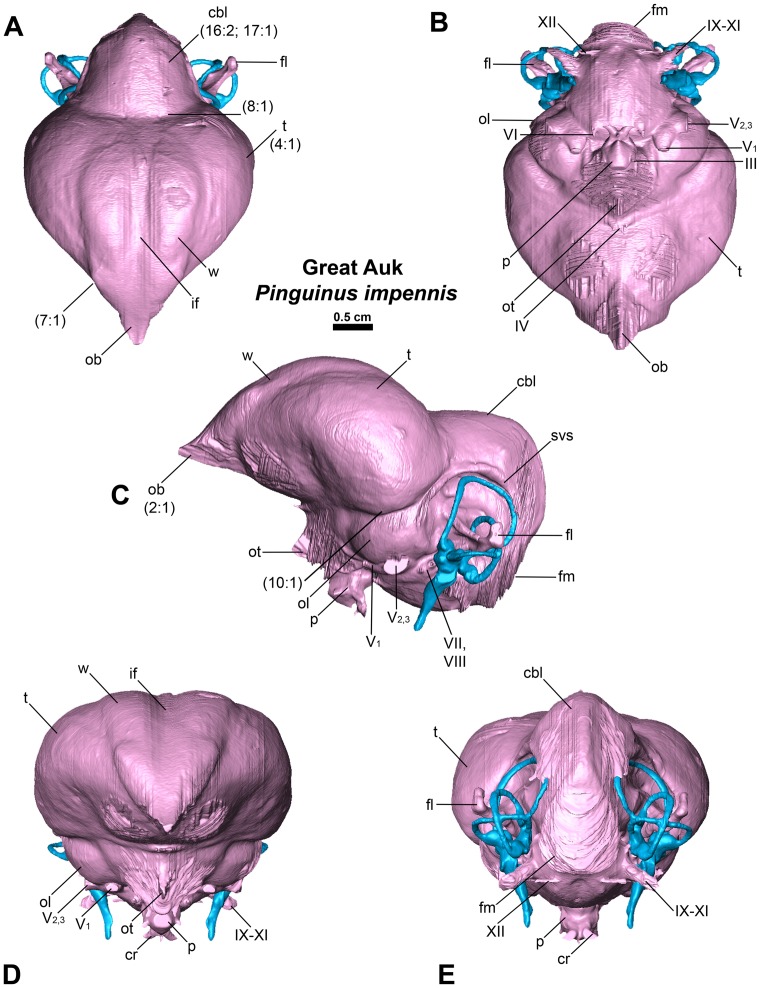
Endocast of the Great Auk *Pinguinus impennis* in dorsal (A), ventral (B), lateral (C), anterior (D), and posterior (E) views. Numbers in parentheses represent character states from Appendix S1.

**Figure 4:**
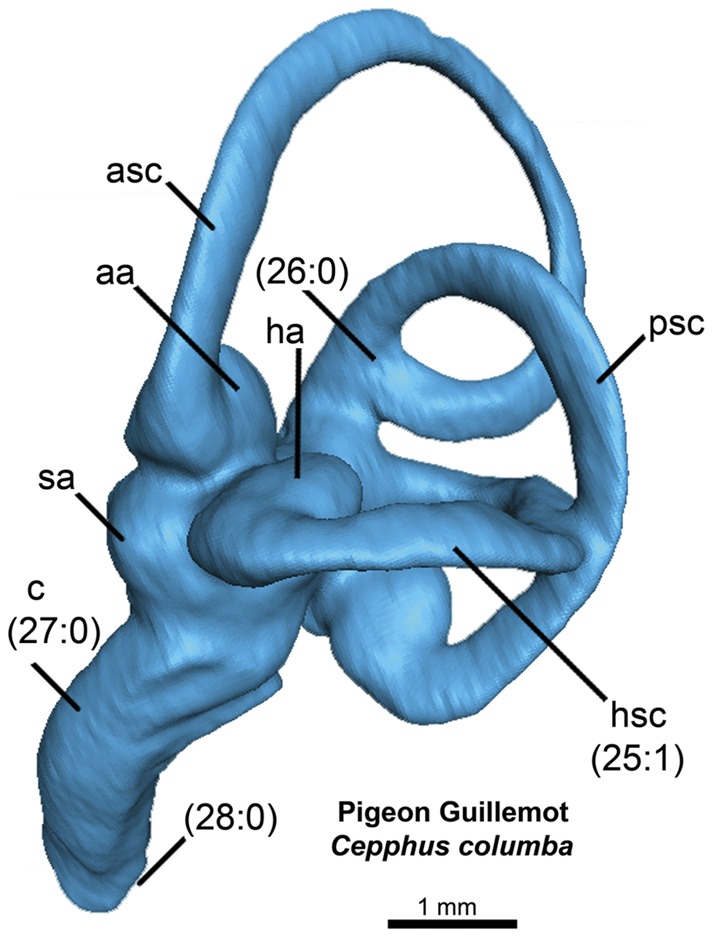
Left endosseous labyrinth of the Pigeon Guillemot *Cepphus columba* in lateral view. Numbers in parentheses represent character states from Appendix S1.

## Materials and Methods

### Computed Tomography Scanning

Reconstructions of brains and other neural tissues in birds (i.e., endocasts) are considered reasonably accurate morphological representations of those structures because the avian brain essentially fills the endocranial cavity [35], [36]. CT scanning of avian skulls at the University of Texas at Austin High-Resolution X-ray Computed Tomography Facility (UTCT) resulted in coronal slices with a high degree of contrast between bone and surrounding matrix (air or lithified sand) and fine scale resolution of osteological details. Digital endocasts of the left and right endosseous labyrinths and the entire endocranial cavity (including major nerve and arterial pathways) were rendered for all 17 newly sampled taxa. Specimens were made available for CT scanning by The American Museum of Natural History, The North Carolina Museum of Natural Sciences, The National Museum of Natural History, and The San Diego Museum of Natural History. Seventeen specimens were scanned at high X-ray energy (200 kV) and resulting serial sections (Fig. 1) were saved as two independent series of 16-bit TIFF files and 8-bit JPEG files (average  =  ∼800 informative slices of the cranial cavity per specimen). CT scan parameters such as slice thickness, field of reconstruction, reconstruction offset, reconstruction scale, and number of slices per rotation varied between scans. Scan resolution (i.e., voxel dimensions) ranged from 0.02734–0.04395 mm (x and y axes) to 0.03135–0.05293 mm (z axis). All raw CT data are archived at UTCT, and details of the scanning parameters for each individual scan are available through UTCT upon request. The 8-bit JPEG files were of high resolution in all extant and extinct species and were used to create 3D computer models of the endocranial cavity and endosseous labyrinths of all 17 sampled taxa using Avizo v6.1 (Mercury Computer Systems, Berlin, Germany; http://www.tgs.com/products/avizo. asp).

**Figure 5:**
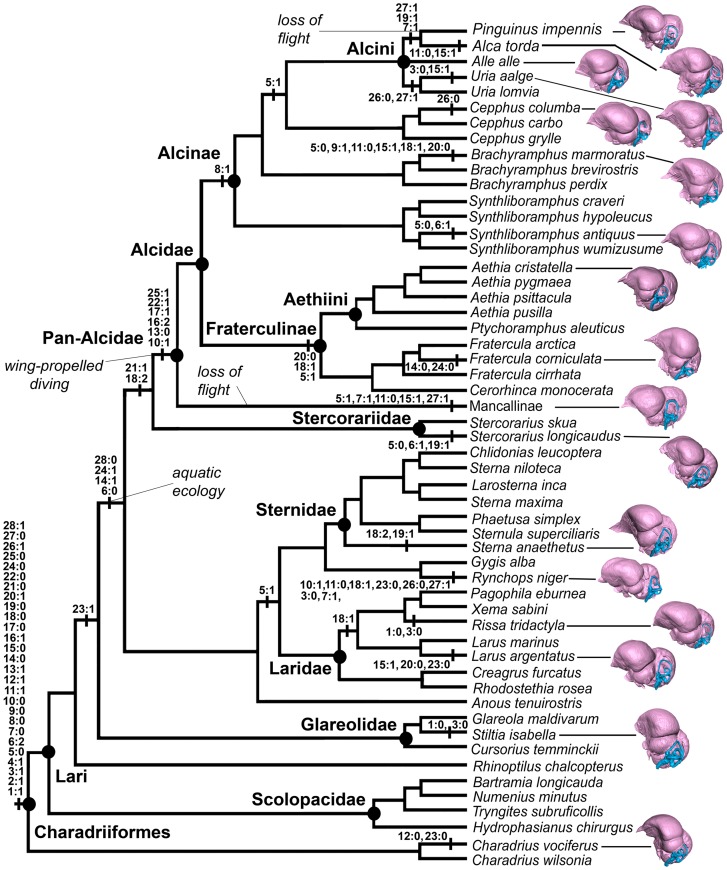
Cladogram of charadriiform relationships showing taxonomic sampling, and all character state changes mapped onto the topology presented by Smith (2011a). *Halcyornis toliapicus*, New Caledonian Crow *Corvus moneduloides*, and Great Horned Owl *Bubo virginianus* are not represented here because they were not included in that analysis (ibid).

**Figure 6:**
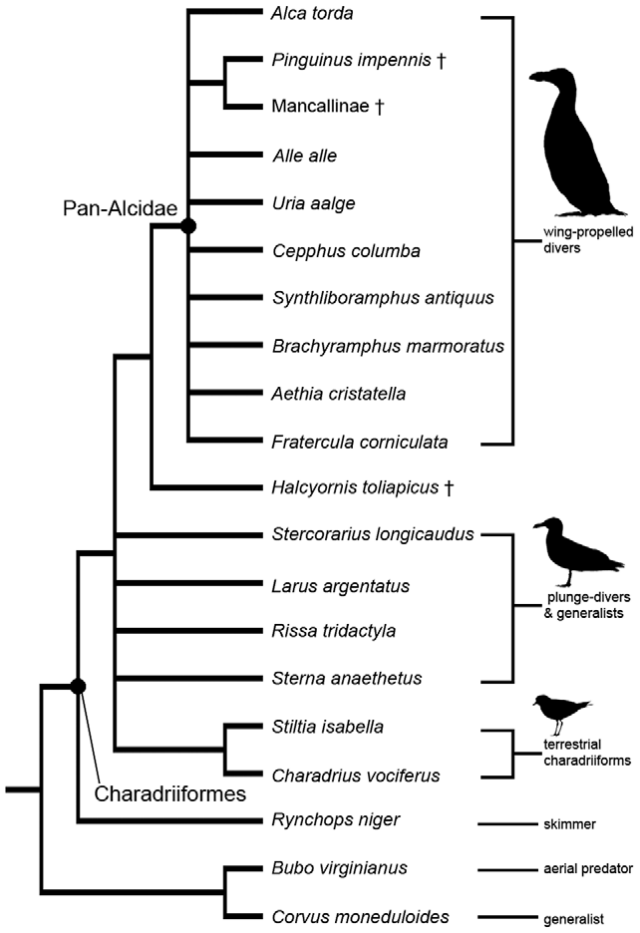
Strict consensus topology resulting from parsimony analysis of 28 endocranial characters (531 MPTs; L: 68; CI  = 0.54; RI  = 0.75; RCI  = 0.41). Note the lack of overall resolution and that the position of *Rynchops niger* at the base of Charadriiformes can likely be attributed to the unique endocranial morphology of that taxon in comparison with other charadriiforms. These results suggest that endocranial characters may not be phylogenetically informative among relatively closely related species in a clade.

### Comparative Methods

Measurements of endocranial volume (ECV) and endosseous labyrinth volume (ELV) were calculated using Avizo v6.1. ECV values include the cranial nerves and a small portion of the carotid rami. ELV values include the cochlear duct and semicircular canals. Inclusion of small portions of the carotid rami do not significantly affect calculated volumes and this method is consistent with previous particle-fill methods. Encephalization quotients (EQ), a comparison of endocranial volume and body mass that controls for allometric effects [35], were calculated for each specimen using the equation EQ  =  ECV/O.137W^0.568^, where W  =  body mass [37]. When body mass data were not recorded for sampled skeletal specimens, values used are the averages cited by Dunning ([38]; Table 3). A species with an EQ >1.0 is considered to have an endocranial volume that is larger than expected for its body mass and EQ values <1.0 are indicative of species with endocranial volumes that are smaller than expected [35]. Because volumes of individual brain regions (e.g., optic lobes) could not be calculated based on exposed surfaces of these structures alone, qualitative comparisons of relative size among taxa were made by scaling endocasts figures to the same vertical dimension and assessing relative size of exposed regions. All specimens CT scanned were adult (isolated fossil skulls assessed based on degree of cranial suture ossification). To assess the morphological consistency of digitally reconstructed endocasts with respect to actual brains, character scorings for one alcid (Dovekie *Alle alle*) and one non-alcid charadriiform (Herring Gull *Larus argentatus*) were confirmed through dissection. Additional specimen information, CT slice images, and endocast images of the Razorbill Auk *Alca torda* (NCSM 20058) are available through UTCT and digimorph.org respectively.

**Figure 7:**
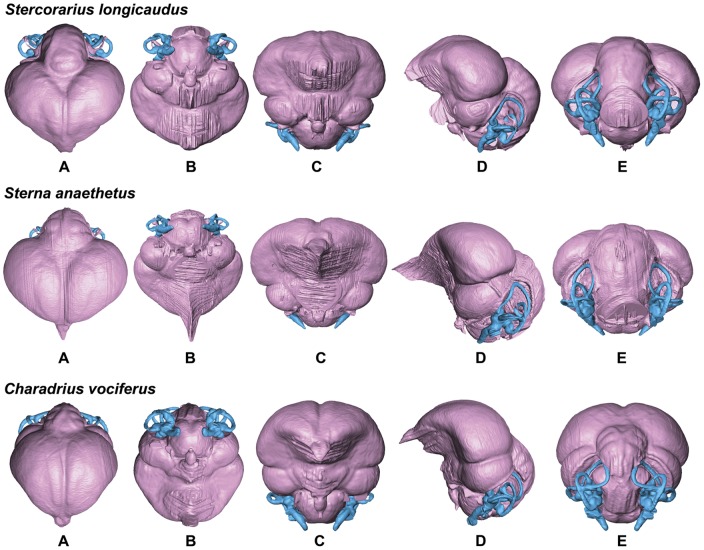
Endocasts of the Long-tailed Skua *Stercorarius longicaudus*, Bridled Tern *Sterna anaethetus*, and Killdeer *Charadrius vociferus* in dorsal (A), ventral (B), anterior (C), lateral (D), and posterior (E) views (not to scale for comparison).

**Figure 8:**
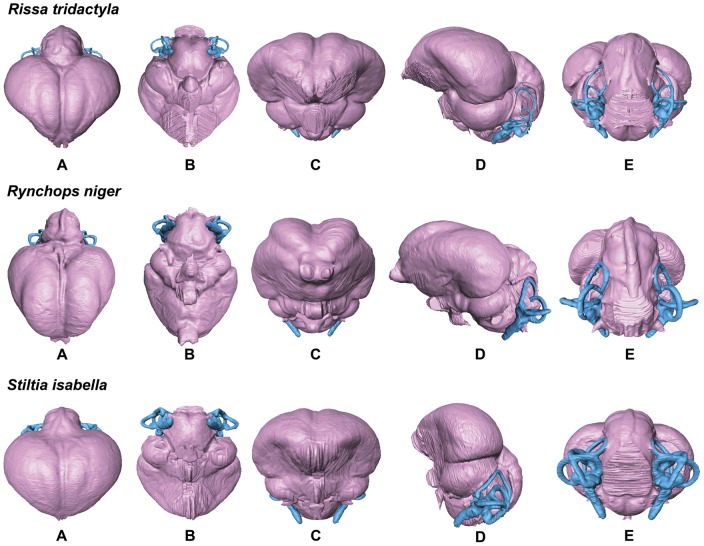
Endocasts of the Black-legged Kittiwake *Rissa tridactyla*, Black Skimmer *Rynchops niger*, and Australian Pratincole *Stiltia isabella* in dorsal (A), ventral (B), anterior (C), lateral (D), and posterior (E) views (not to scale for comparison).

**Figure 9:**
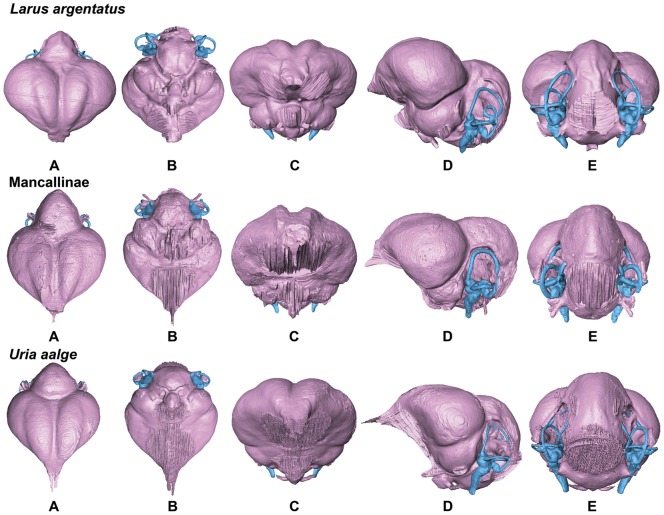
Endocasts of the Herring Gull *Larus argentatus*, Mancallinae sp., and Common Murre *Uria aalge* in dorsal (A), ventral (B), anterior (C), lateral (D), and posterior (E) views (not to scale for comparison).

**Figure 10:**
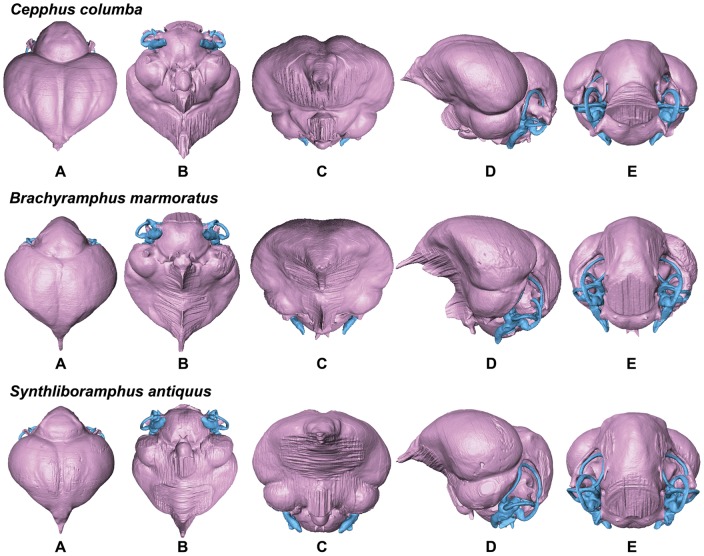
Endocasts of Pigeon Guillemot *Cepphus columba*, Marbled Murrelet *Brachyramphus marmoratus*, and Ancient Murrelet *Synthliboramphus antiquus* in dorsal (A), ventral (B), anterior (C), lateral (D), and posterior (E) views (not to scale for comparison).

**Figure 11:**
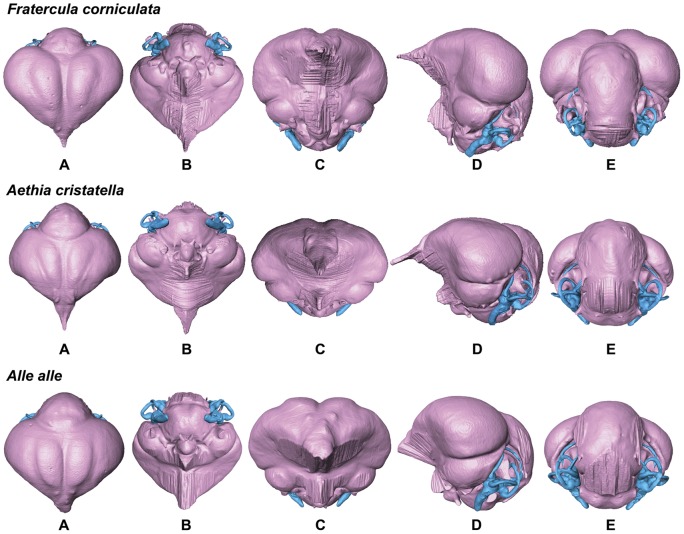
Endocasts of Horned Puffin *Fratercula corniculata*, Crested Auklet *Aethia cristatella*, and Dovekie *Alle alle* in dorsal (A), ventral (B), anterior (C), lateral (D), and posterior (E) views (not to scale for comparison).

Because species do not represent independent data points in statistical analyses [39], regression analyses of endocranial volume, endosseous labyrinth volume and body mass were performed using a phylogenetic generalized least squares model (PGLS) to explore the relationship between these variables using the software package caper [40] in R v2.15.0 [41]. All data were natural log transformed to normalize distribution and variance. We estimated Pagel's λ to assess phylogenetic signal in the data based on the topology of Smith (2011a) and branch lengths computed using Grafen's method [42] because that topology was based on analysis of combined data.

### Taxon Sampling and Phylogenetic Approach

Taxa were chosen to sample all major charadriiform sub-clades (Table 1). Denser sampling within Pan-Alcidae reflects a particular study focus on potential shifts in sensory systems associated with wing-propelled diving taxa. The extinct Pan-Alcidae stem lineage of Alcidae is represented by Mancallinae sp. from the Middle to Late Pliocene (3.6–1.8 Ma) San Diego Formation of the eastern Pacific Ocean basin (SDSNH 25373; [28]). However, morphological comparisons were also made between the 17 newly-generated charadriiform endocasts and 13 additional charadriiform taxa with variable ecologies (terrestrial foragers, aerial generalists, aerial plunge divers and an aerial surface skimmer) from previously published sources [2], [7]–[9], [13], [17], [18], [23], [43], [44] (Table 1).

**Figure 12:**
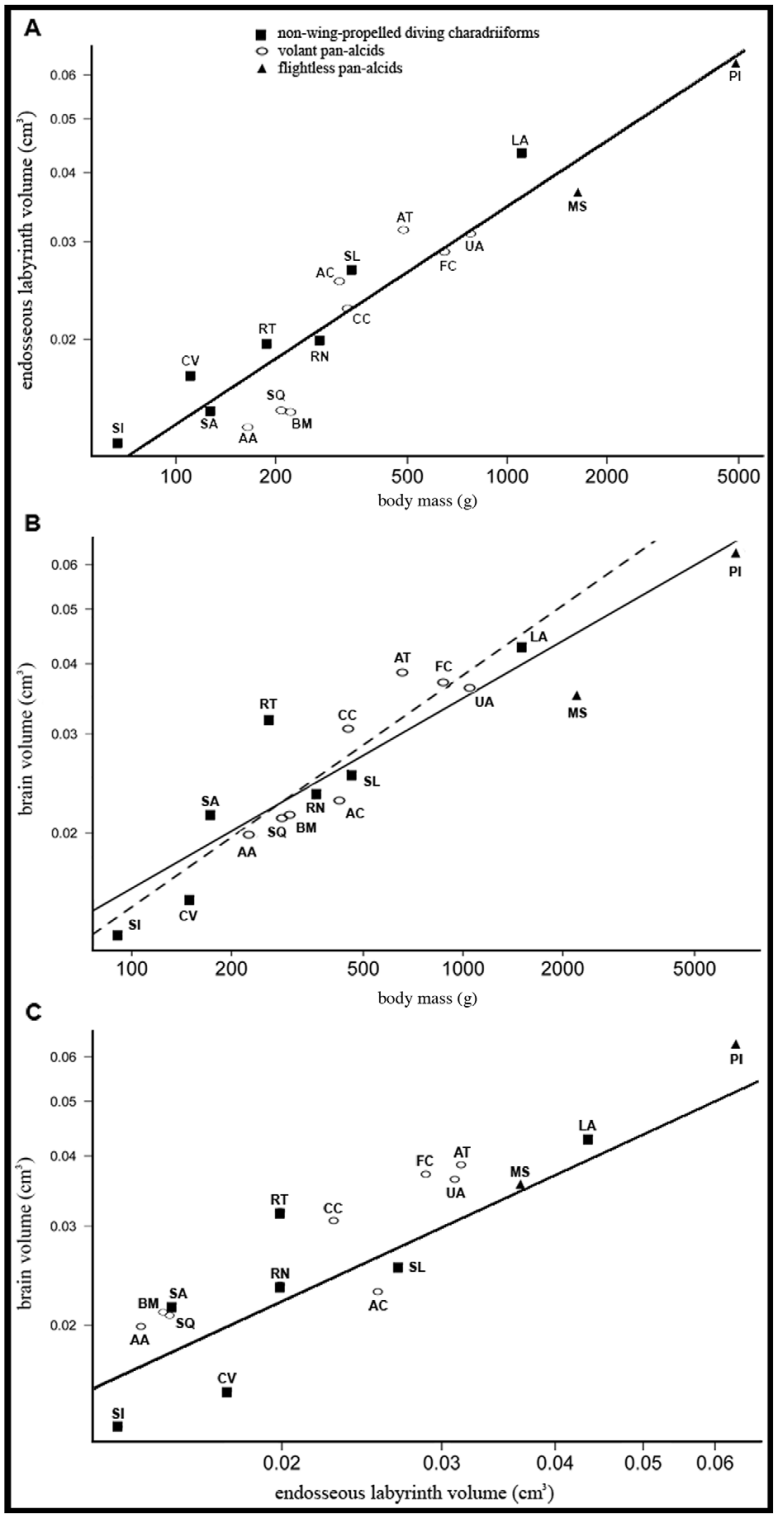
Scatterplots showing the relationship between endosseous labyrinth volume and body mass (A; r^2^ = 0.88), endocranial volume and body mass (B; r^2^ = 0.83, β = 0.510±0.058 s.e.), and between endocranial volume and endosseous labyrinth volume (C; r^2^ = 0.82). All values (Table 3) were natural log transformed and are plotted on scales of raw values. The dashed regression line in B shows the change in slope that resulted when data for flightless species *Pinguinus impennis* and Mancallinae sp. were removed (re-calculated values: r^2^ = 0.88; β = 0.626±0.085 s.e.). Taxonomic abbreviations: *Aethia cristatella* (AC), *Alca torda* (AT), *Alle alle* (AA), *Brachyramphus marmoratus* (BM), *Cepphus columba* (CC), *Charadrius vociferus* (CV), *Fratercula corniculata* (FC), *Larus argentatus* (LA), Mancallinae sp. (MS), *Pinguinus impennis* (PI), *Rissa tridactyla* (RT), *Rynchops niger* (RN), *Stercorarius longicaudus* (SL), *Sterna anaethetus* (SA), *Stiltia isabella* (SI), *Synthliboramphus antiquus* (SQ), *Uria aalge* (UA).

To assess endocranial differences between Charadriiformes and other birds, comparisons were also made with a broad sample (*n = *19) of previously published endocasts of non-charadriiform birds of highly variant ecologies [2], [7], [8], [13], [17], [18], [23], [45](Table 2). These outgroup comparisons included species of owls and a crow because Strigiformes and Passeriformes have been recovered as comparatively close outgroups of Charadriiformes (i.e., as parts of clade G of [46]). In addition to these taxa, an array of outgroup comparisons were also made with basal neognaths (i.e., Galloanseres) and a small sample of paleognaths (ratites). Because the transition to wing-propelled diving was one of the main foci of this study, comparisons were also made with non-charadriiform divers such as penguins and with previously published endocasts of extinct species with proposed ecologies similar to some of the included charadriiform taxa (Table 2).

**Figure 13:**
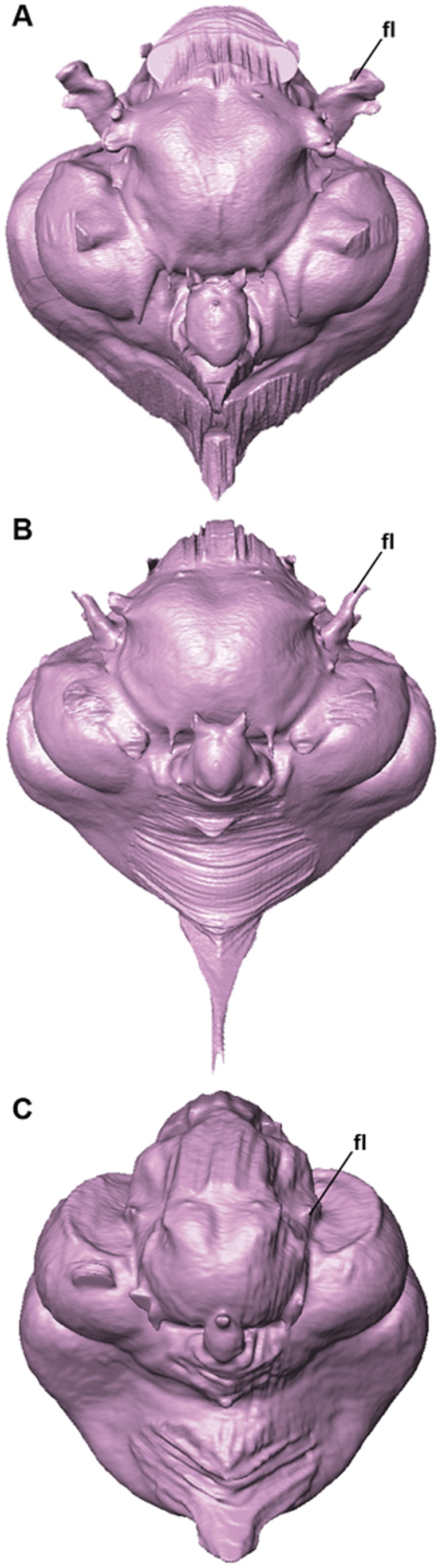
Ventral views of endocranial endocasts of the Pigeon Guillemot *Cepphus columba* (A), Crested Auklet *Aethia cristatella* (B), and Black-legged Kittiwake *Rissa tridactyla* (C) showing differences in the relative size and morphology of the floccular lobe.

Endocranial cavity and inner ear variation among the 17 newly CT scanned charadriiforms, two outgroup taxa and *Halcyornis toliapicus*, an extinct taxon of proposed charadriiform affinities was represented by 28 discrete morphological characters (Appendices S1 & S2). Additional charadriiforms and outgroup taxa (Tables 1, 2) were excluded from character analyses because figures of previously published taxa lacked comparable detail when compared with the digitally rendered endocasts (i.e., nerves, arteries, floccular lobes, and endosseous labyrinths not visible or not reconstructed). Character numbers and character states (Figs. 2, 3, 4) follow descriptions in the text (e.g., 2∶1 refers to character 2, state 1). Phylogenetically contextualized inferences were based on the hypothesis of charadriiform relationships proposed by Smith (2011a). The results of that analysis are based upon a combined analysis of morphological characters and molecular sequence data and are largely congruent with the results of recent analyses based only on molecular sequence data [27], [30]. Differences in hypothesized charadriiform relationships between the results of Smith (2011a) and the molecular sequence-based analyses of Baker et al. (2007) and Pereira and Baker (2008) are restricted to the positions of *Synthliboramphus* in Alcidae, and the positions of *Anous tenuirostris, R. niger*, and *Gygis alba* among non-alcid charadriiforms (discussed by Smith 2011a). All unambiguous charadriiform endocranial character state changes were mapped. Character differences between Charadriiformes and the sampled outgroup taxa (Great Horned Owl *Bubo virginianus*, New Caledonian Crow *Corvus moneduloides*) are inferred at the base of crown Charadriiformes because only charadriiforms were included in the analysis of Smith (2011a). Consistency indices for mapped characters were assessed in Mesquite v2.75 [47]. Endocranial characters were also analyzed to assess congruence between relationships recovered using endocranial data alone and the phylogeny recovered by Smith (2011a). It should be noted that this analysis was used descriptively, and the phylogenetic utility of endocranial characters at the interspecies level within a clade has not been proposed nor previously investigated. The degree to which these characters may be linked to broader change in brain shape across phylogeny is also unknown. The phylogenetic analysis was performed using a branch and bound search strategy in PAUP* v4.0a122 [48]. All characters were equally weighted and minimum length branches  = 0 were collapsed. The tree was rooted with the ‘landbird’ outgroup taxa *Bubo virginianus* and *Corvus moneduloides*.

**Figure 14:**
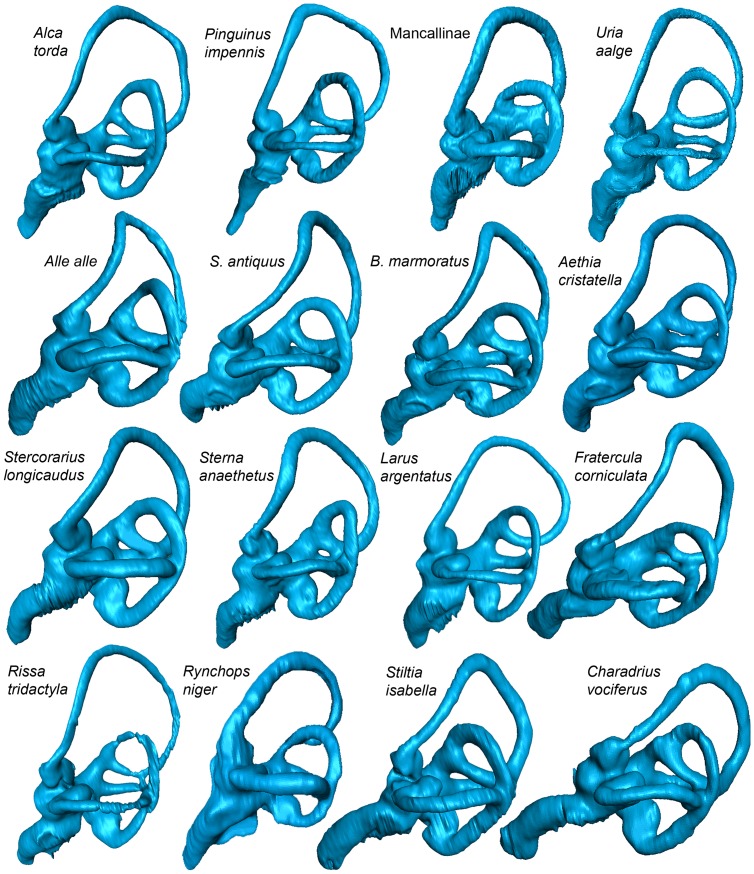
Left endosseous labyrinths of sampled taxa in lateral view (not to scale for comparison). Note that compression of the semicircular canals is often difficult to visualize without being able to manipulate reconstructed canals in 3-D.

**Figure 15:**
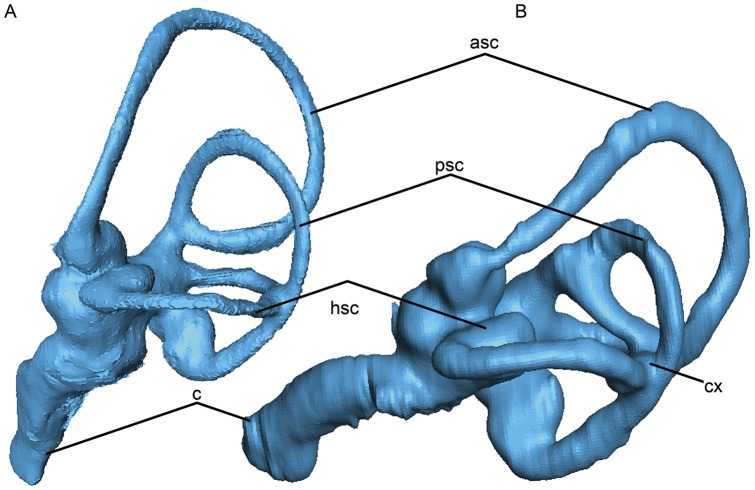
Left endosseous labyrinths of the aquatic, flightless Great Auk *Pinguinus impennis* (A) and the terrestrial foraging, volant Killdeer *Charadrius vociferus* (B) in lateral view (not to scale for comparison). Note the differences in the orientation of the labyrinths (based on horizontal semicircular canal), compression of the semicircular canals (i.e., elliptical versus rounded shape), overall thickness of canals, curvature of the cochlear duct, shape of the distal end of the cochlear duct, and participation of the anterior semicircular canal in the common crus.

### Anatomical Abbreviations

English equivalents of the anatomical terminology for the central nervous system summarized by Breazile and Kuenzel [49] are used: aa, anterior ampulla; asc, anterior semicircular canal; c, cochlea; cbl, cerebellum; cc, cranial cavity; cf, cerebellar fissures; cr, carotid ramus; cx, common crus; el, endosseous labyrinth; f, frontal; fl, cerebellar flocculus; fm, foramen magnum; ha, horizontal ampulla; hsc, horizontal semicircular canal; if, interhemispherical fissure; ob, olfactory bulb; ol, optic lobe; os, occipital sinus; ot, optic tract; p, pituitary gland (hypophysis); psc, posterior semicircular canal; pts, paratympanic sinus; r, rhombencephalon; sa, sacculus; svs, semicircular vein sulcus; t, telencephalon; w, wulst (sagittal eminence), III–XII, cranial nerves III–XII.

**Figure 16:**
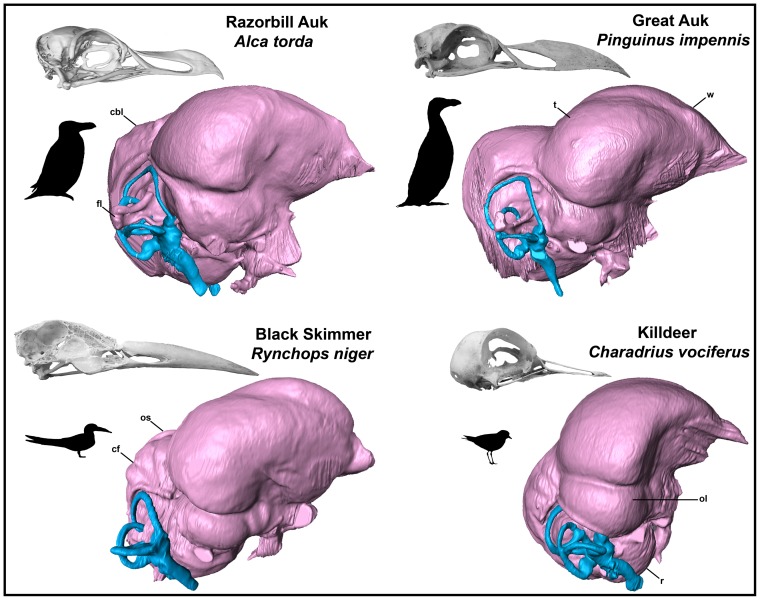
Endocranial endocasts of the Razorbill Auk *Alca torda* (A), Great Auk *Pinguinus impennis* (B), Black Skimmer *Rynchops niger* (C), and Killdeer *Charadrius vociferus* (D) in right lateral view (not to scale for comparison). These four endocasts illustrate the morphological differences between aquatic versus terrestrially foraging charadriiforms, between *R. niger* and all other charadriiforms, between wing-propelled diving pan-alcids and other charadriiforms, and between volant and flightless pan-alcids. Note the differences in the dorsal projection of the wulst, the relative size of the optic lobe as compared to the telencephalon, the relative length of the cerebellum as compared to that of the telencephalon, the relative size of floccular lobes, absence or presence of cerebellar fissures and occipital sinuses, and the difference in the relative orientation of the telencephalon, optic lobe, and rhombencephalon between *Charadrius vociferus* and other figured species. Note that the lateral surface of the skull of *R. niger* (C) has been cut away to show the position of the cranial cavity.

### Institutional Abbreviations

AMNH–American Museum of Natural History, New York, NY, USA; EB–Ehime University, Ehime, Japan; EPSM-AV–Ehime Prefectural Science Museum, Ehime, Japan; KU–University of Kansas Natural History Museum, Lawrence, KS, USA; LB–Auckland Museum, Aukland, New Zealand; MLP–Museo de La Plata, La Plata, Argentina; NHMUK–Natural History Museum, London, England; NCSM–North Carolina Museum of Natural Sciences, Raleigh, NC, USA; NSM PO–National Museum of Nature and Science, Paleontology Osteological Collection, Tokyo, Japan; OUVC–Ohio University Vertebrate Collections, Athens, OH, USA; SDSNH–San Diego Natural History Museum, San Diego, CA, USA; TCWC–Texas Cooperative Wildlife Collection, Texas A&M University, College Sation, TX, USA; USNM–Smithsonian Institution, National Museum of Natural History, Washington, D.C., USA; UTCT–University of Texas at Austin High Resolution X-ray Computed Tomography Facility, Austin, TX, USA.

## Results and Discussion

Our results show that Charadriiformes display a higher degree of endocranial variation (morphologically and volumetrically) than previously studied clades of birds. These results are congruent with a previous study of cerebrotype variation that found charadriiforms to be the most disparate among sampled parameters [1], and with previous studies that documented substantial endocranial variability among a small sample of Charadriiformes [44], [50]. However, as mentioned above, previous studies of endocranial variation within avian clades are few [13], [15]–[17] and comparably dense taxonomic sampling in other clades that display a range of ecological attributes (e.g., Anatidae) might identify additional variation. As we describe below, many of the differences in charadriiform endocranial morphology appear to correlate with phylogeny and derived ecologies (e.g., wing-propelled diving clade Pan-Alcidae have distinct endocranial morphology). Below, we first review the optimized distribution of evaluated endocranial characters and report several proposed (i.e., tentative owing to limited outgroup sampling) apomorphies of Charadriiformes and endocranial morphologies apparently particular to wing-propelled divers. Next, we present volumetric comparisons of charadriiform endocrania and discuss potential correlations with a suite of sensory and ecological attributes. Discussion of morphological variation of distinct sub-regions in the charadriiform brain, relative orientation of charadriiform endocranial regions, and inner ear variation follows. Finally, we discuss the extreme endocranial modifications of the Black Skimmer, modifications that make this taxon unique among sampled charadriiforms.

### Endocranial Apomorphies of Charadriiformes

Mapping of endocranial character changes on a phylogeny of charadriiform relationships allowed for inferences to be made regarding acquisition of morphological changes of the endocranium in wing-propelled diving pan-alcids relative to other charadriiforms and in charadriiforms relative to outgroup taxa (Fig. 5). A large suite of character state changes separates Charadriiformes from sampled outgroup taxa (Fig. 5) but further outgroup sampling or iterative outgroup swapping would likely indicate many of these characters show more homoplasy and would be ambiguously optimized. The 28 endocranial characters had consistency indices (CI) ranging from 0.25–1.0. Bifurcation of the olfactory bulb (3∶0/1) and the relative length of the telencephalon as compared to the cerebellum (15∶0/1) showed the most homoplasy, with variation among Charadriiformes, among Pan-Alcidae, and in the outgroup. Twelve of the 28 characters had a CI  = 1.0 (characters 2, 4, 8, 9, 12, 13, 16, 17, 21, 22, 25, 28). Phylogenetic analysis of the endocranial dataset resulted in a nearly completely unresolved topology (Fig. 6). The first named fossil bird, the isolated cranial holotype of *Halcyornis toliapicus* was recovered as the sister taxon to an unresolved yet monophyletic Pan-Alcidae. The systematic position of *H. toliapicus* has not been previously evaluated in a phylogenetic analysis. The Black Skimmer was placed as the sister taxon to the rest of Charadriiformes, a result that conflicts with the placement of that taxon in previous analyses in which it has been recovered as the sister taxon to Laridae or Sternidae but reflects the striking disparity in endocranial morphology seen in this taxon. [27], [28], [51]. Based on these results, avian endocranial characters have the potential to be misleading in a phylogenetic context and should be used with caution until the relative contributions of phylogeny and behavior-based morphological groupings can be further evaluated through denser character and taxon sampling.

In comparison with sampled outgroup taxa, four proposed apomorphies characterize the endocranium of charadriiforms. The olfactory bulbs of all sampled charadriiforms are positioned more dorsally (2∶1) than those of the sampled outgroup taxa the Great horned Owl and the New Caledonian Crow. However, reconstructions of charadriiform olfactory bulbs were tentative in some cases (e.g., Great Auk) owing to a partial lack of bony architecture in the corresponding region of the skull, and therefore, identified morphological variation was primarily limited to the relative size and position of this structure. The telencephalon occupies the majority of the dorsal surface of the brain in Aves and was found to vary substantially among the taxa surveyed (Figs. 2, 3, 7, 8, 9, 10, 11). The telencephalon of all sampled charadriiforms was anteriorly tapered (4∶1) in comparison with the conspicuously rounded telencephalic hemispheres of the Great Horned Owl and the New Caledonian Crow. However, an intermediate condition in which the anterior telencephalon is slightly less tapered is present in the Eurasian Woodcock *Scolopax rusticola*, Northern Lapwing *Vanellus vanellus*, Grey Plover *Pluvialis squatarola*, Egyptian Plover *Pluvianus aegyptius*, and European Golden Plover *Pluvialis apricaria* [44]. The tapered condition characteristic of charadriiforms is likely pleisiomorphic for Aves as it is also present in tinamous [13], [18] and anseriforms such as the Northern Screamer *Chauna chavaria* and the Mallard *Anas platyrhynchos*. In contrast, the North Island Giant Moa *Dinornis novazealandiae*, and the Rock Dove *Columbia livia* display the more evenly rounded, derived condition [13], [18], [43]. Additionally, the cerebella of Charadriiformes were relatively wider (16∶1/2) than those of compared outgroup taxa. Finally, the optic tract of sampled charadriiforms is relatively larger than those of sampled outgroup taxa (22∶0/1).

Four endocranial characters support an ‘aquatic charadriiform clade’ including Pan-Alcidae, Stercorariidae, Sternidae, Laridae, and Rynchopidae. This clade is characterized by a wulst that extends from the olfactory bulb to the cerebellum (6∶0), <50% of the posterior margin of the telencephalon in contact with the cerebellum (14∶1), horizontally oriented long axis of the brain (24∶1), and tapered rather than swollen distal tip of the cochlear duct (28∶0). Given a hypothesis of extant and extinct charadriiform relationships based on analysis of morphological and molecular sequence data (Fig. 5), the sister taxon relationship between Pan-Alcidae and Stercorariidae is supported by two unambiguously optimized endocranial apomorphies (elongate cerebellar flocculus, 18∶2; inconspicuous rhombencephalon sulcus, 21∶1).

The affinities of *Halcyornis toliapicus* Koenig 1825, have remained uncertain even though *H. toliapicus* has had the longest history of study of any fossil bird [52]. Uncertainty surrounding this species is not surprising given that it is known only from an incomplete cranium (NHMUK A130). *H. toliapicus* has been previously allied with charadriiforms, coraciiforms, and stem psittaciforms [9], [52]–[56]. When compared to endocranial endocasts of other Eocene birds Walsh and Milner (2011b) concluded that *H. toliapicus* was most morphologically similar to the larid sampled. They noted the relatively large olfactory bulb of *H. toliapicus*. Indeed, the Herring Gull *Larus argentatus* is the only charadriiform taxon sampled herein with an olfactory bulb approaching the size of that of *H. toliapicus* (Fig. 9) and the distinct cerebellar fissures and occipital sinus of *H. toliapicus* also are closer to non-alcid charadriiforms such as the Herring Gull. However, as discussed below, features of the inner ear are consistent with pan-alcid affinities for the taxon.

### Endocranial Characteristics of Pan-Alcidae

Six endocranial characters are interpreted as apomorphies of Pan-Alcidae relative to non-wing-propelled diving charadriiforms. These apomorphies include a curved rather than straight contact between the telencephalon and the dorsal optic lobe (10∶1), occipital sinus not clearly visible along dorsal surface of the cerebellum (13∶0), relatively wide cerebellum (16∶2), indistinct cerebellar fissures (17∶1), contact between the optic tract and the pituitary gland (22∶1), and compression of the semicircular canals of the endosseous labyrinth (25∶1).

The absence of distinct cerebellar fissures on the outer surface of the endocasts of pan-alcids is in stark contrast to the morphology of other sampled taxa. The lack of cerebellar fissures on the endocasts is likely a result of a thicker layer of meningeal tissue that obscures those features in these taxa, not an indication that these taxa lack cerebellar fissures. Alternatively, the occipital sinus may be broad and dorsoventrally compressed, thus obscuring the cerebellar fissures along the dorsal cerebellum. Correlates of indistinct cerebellar fissures in Aves remain to be investigated. This condition is also seen in some other diving birds including penguins (e.g., Chinstrap Penguin *Pygoscelis antarctica*; [17], [45]), the Common Loon *Gavia immer* [17], Great Cormorant *Phalacrocorax carbo* [43], and the extinct bird *Enaliornis barretti* [57]. Previous studies found that cerebellar morphology is phylogenetically conserved, and that overall, charadriiforms have relatively large and complexly folded cerebella [58], [59]; however, those studies did not include any alcids. Although links between cerebellar morphology, cognitive ability, and behavioral attributes such as motor control and sensory integration have been proposed [58], [59], the functional significance of cerebellar fissure morphology remain untested. The restriction of indistinct cerebellar fissures to Pan-Alcidae among Charadriiformes, and the lack of these structures in endocasts of other diving birds make it tempting to correlate this characteristic with diving. Nonetheless, a broader taxonomic sample including the extinct Plotopteridae and diving anseriforms (e.g., *Aythya*) is needed to further evaluate this hypothesis.

Another feature optimized as an apomorphy of Pan-Alcidae is contact between the optic tract and the pituitary gland (22∶1). The optic tract is separated from the pituitary gland in other sampled charadriiforms and some other birds (e.g., *Struthio camelus* Ostrich, *Rhea americana* Rhea, *Tyto alba* Barn-owl, and the Rock Dove [7], [13]). Because the optic tract also contacts the pituitary gland in penguins, it is once again, tempting to correlate this feature with wing-propelled diving. However, as with the indistinct cerebellar folds that are optimized as apomorphies of Pan-Alcidae and also seen in penguins, further study is required to verify this potential relationship. These characters may be related to the size of the optic tract but could not be evaluated in the outgroup because the optic tract is substantially smaller in those taxa.

Within Pan-Alcidae, a single endocranial apomorphy supports Alcinae monophyly (contents of Alcinae include *Alca, Pinguinus*, *Uria, Alle, Cepphus, Brachyramphus*, and *Synthliboramphus*; 8∶1, curved junction between telencephalon and cerebellum in dorsal view), and three endocranial characters are optimized as apomorphies of Fraterculinae (contents of Fraterculinae include *Fratercula, Cerorhinca, Aethia*, and *Ptychoramphus*; 5∶1. 18∶1, 20∶0; Fig. 5). Additional description of charadriiform endocranial variation can be found in Appendix S3.

### Charadriiform Endocranial Size Variation

Phylogenetically informed comparisons revealed that, as expected, endocranial volume and endosseous labyrinth volume, endocranial volume and body mass, and endosseous labyrinth volume and body mass appear positively correlated (Fig. 12; Table 3). Data points representing individual species are relatively tightly fitted along the regression lines of the plots of body mass versus endosseous labyrinth volume, and endosseous labyrinth volume versus endocranial volume (Fig. 12). However, the spread of data points in the body mass versus brain volume plot is greater. Removal of the flightless Great Auk and Mancallinae sp., which showed significantly smaller EQ values (see discussion below), resulted in a much tighter fit of volant species along a re-calculated regression of body mass versus endocranial volume. The endocranial measurement data displayed no significant phylogenetic signal (i.e., Pagel's λ values near zero).

Relative endocranial volume of pan-alcids in the present sample is quite variable, including the smallest (Mancallinae sp., EQ = 0.488; Table 3) and second largest (Razorbill Auk, EQ  = 1.131) values in the charadriiform sample. However, the average encephalization of sampled pan-alcids is smaller (EQ  = 0.782) than that of other sampled charadriiforms (EQ  = 0.863). Increased encephalization has been linked to species richness, range expansion, and environmental flexibility [60]–[63], and recent evidence suggests that the Razorbill Auk has adapted rapidly to climate change [64]. Subsequently, environmental adaptability was recently raised as a potential contributing factor in the considerable species richness (*n* = 7 spp.) documented in *Alca* during the Pliocene [65]. However, the species exemplar from the most species-rich extant alcid clade, the auklets (Aethiini; represented by the Crested Auklet *Aethia cristatella*), displayed the lowest encephalization quotient among volant alcids (Table 3). Further sampling is needed to determine if these apparently contrasting trends are real or are perhaps an artifact of limited sampling within these clades.

The hypothesis that flightlessness leads to smaller relative endocranial volume across all Aves [66] was not supported by the results of Iwaniuk et al. (2004). However, the results of that study [11] showed that two wing-propelled diving taxa that have lost aerial flight, the Great Auk and the Emperor Penguin *Aptenodytes forsteri*, were two of only four species that displayed a statistically significant reduction in endocranial volume when compared to closely-related volant species in Aves [11], [34]. Our results also indicate that the endocranial volume of the Great Auk is relatively smaller than that of other charadriiforms (Table 3; Fig. 12). Furthermore, the endocranial volume of the flightless Mancallinae sp. is the smallest in our sample. Unlike the flightless condition in ratites, loss of aerial flight is not a degenerative condition in wing-propelled divers, but rather an extreme specialization for underwater locomotion [34]. The relatively smaller brains of flightless taxa such as the Great Auk may be a trade-off between neural and somatic growth [11] and might be related to differences in pressure (i.e., water pressure at depth) experienced by diving birds that are not encountered by large terrestrial flightless taxa such as ratites. With the additional recovery of a relatively smaller endocranial volume in Mancallinae, additional scrutiny of potential explanations of this pattern in wing-propelled diving taxa is merited. The placement of Mancallinae as the sister taxon to the Alcidae crown clade [28] might also suggest and overall increase in encephalization in the crown clade; however, additional sampling of Mancallinae and charadriiform fossils outside of Pan-Alcidae are needed to test this hypothesis.

With respect to developmental strategies in birds along the altricial to precocial spectrum, altricial species have been found to possess relatively larger endocranial volumes than precocial species [37], [66], [67]. This proposed relationship is not apparent in the EQ values of sampled charadriiforms (Tables 1, 2, 3). The EQ of semi-precocial charadriiform species were all >0.74, with a range of 0.741 in the Ancient Murrelet *Synthliboramphus antiquus* to 1.131 in the Razorbill Auk. In contrast, the EQ of precocial species was lower, ranging from 0.646 in the Killdeer *Charadrius vociferus* to 0.998 in the Pigeon Guillemot *Cepphus columba*. Although there is overlap in EQ ranges, the semi-precocial charadriiform average (EQ average  = 0.989) is significantly higher than that of precocial charadriiforms (EQ average  = 0.756; Kruskal-Wallis rank sum test: χ^2^_1_ = 6.125, p = 0.013). Values for Mancallinae sp. and the Great Auk were excluded from this comparison because details of the breeding biology of these extinct taxa are not known. These data do not necessarily conflict with the hypothesis of relatively larger endocranial volumes in altricial species; however, these data suggest that the relationship between developmental strategy and endocranial volume is more complex in Charadriiformes than across Aves as a whole.

There is no clear trend in the relationship between relative endocranial volume, dive depth, and body mass in this sample of charadriiforms. The relatively greater body mass of the Great Auk (∼5000 g) and the Emperor Penguin (up to 45000 g) in relation to smaller members of their clades such as the Razorbill Auk (524–890 g) and the Little Penguin *Eudyptula minor* (∼1000 g) respectively, was proposed to be related to increased depth of dives by these larger species [11]. Unfortunately, details of dive depth for the recently extinct Great Auk are not known. However, the encephalization quotient of the largest and most deeply diving extant alcid, the Thick-billed Murre *Uria lomvia* (940 g, 210 m) is lower than that of three smaller alcids with shallower maximum dive depths (Horned Puffin *Fratercula corniculata* 612g, 40 m; Razorbill Auk 120 m; Pigeon Guillemot 450–550 g, 20 m; Table 3; dive depths and body mass data based on those compiled by Smith 2011b). Although these data do not support positive correlation between dive depth and increased encephalization in Alcidae, greater dive depth may be positively correlated with somatic growth as suggested by Iwaniuk et al. (2004).

### Variation of Distinct Sub-regions in the Charadriiform Brain

The relative size of different regions of the vertebrate brain is proportional to the relative importance of that particular neural region to any given species [35], [68]. Discussion of the relative size or position of the wulst, or sagittal eminence is a common theme in the literature on avian brains because this structure has been variably linked with sociality, vision, and somatosensory perception [1], [20], [22], [69]. Specifically, the anterior wulst receives somatosensory information from body, limbs, head and neck, and is also associated with visual information processing such as contour-based form perception (i.e., prey identification; [70], [71]) and stereopsis [12], [13], [50], [72]). Furthermore, anterior restriction of the wulst has been proposed as a correlate of decreased dependence on visual stimuli [50], [73]. In the present sample, there is considerable variation in the dorsal and anterolateral expansion of the wulst as well as in its anteroposterior extent (see Appendix S1, characters 5–7; Figs. 2, 3, 7, 8, 9, 10, 11). Characters of wulst morphology display considerable homoplasy within Pan-Alcidae and other sampled Lari, including some species which, are otherwise similar in feeding, locomotor and other ethological attributes (e.g., *Aethia* and *Brachyramphus*). Among sampled Pan-Alcidae, the wulst of the Marbled Murrelet *Brachyramphus marmoratus* and the Ancient Murrelet are less dorsally expanded (5∶0) than those of other pan-alcids. Additionally, the wulst extends from the olfactory bulb to the cerebellum in most sampled charadriiforms (e.g., Razorbill Auk), whereas the wulst is positioned posteriorly (6∶1) in the Ancient Murrelet among Alcidae and in the Long-tailed Skua and Eurasian Woodcock [44] among non-alcid charadriiforms. Furthermore, the only two terrestrial foraging charadriiform taxa sampled are also the only two taxa that possess wulsts that are positioned midway between the olfactory bulb and the cerebellum (6∶2). Finally, the anterior portion of the wulst is laterally expanded (7∶1) in the flightless taxa the Great Auk and Mancallinae sp., as well as in the volant taxa the Razorbill Auk and the Black Skimmer.

The flightless Mancallinae sp. and Great Auk have relatively smaller optic lobes than other sampled pan-alcids (11∶0; Figs. 2, 3, 7, 8, 9, 10, 11). The relatively smaller size of the optic lobe in these flightless taxa is likely an example of convergence, as these species are not hypothesized to be closely related within Pan-Alcidae ([28]; Fig. 5). Flightless wing-propelled penguins also appear to have relatively small optic lobes in comparison with closely related taxa such as loons and tubenoses [17]. In contrast to the apparent convergence among flightless wing-propelled divers, a trend in the relative size of the optic lobe in flightless terrestrial paleognaths is not apparent. *Struthio camelus* (ostrich), *Dromaius novaehollandiae* (emu), and *Rhea americana* (rhea) have relatively large optic lobes in comparison with nocturnal kiwi (e.g., *Apteryx mantelli*) and the extinct moa (e.g., *Dinornis novazealandiae*) [7], [13], [40], [42]. Because the relative size of different neural regions is linked with the relative importance of that region [35], [68], one potential explanation for the reduced size of the optic lobe in the Great Auk and Mancallinae sp. may be related to the lack of optical input associated with the complexities of aerial takeoff, maneuverability, and landing. However, because the optic lobe also receives sensory input from auditory and somatosensory sources, establishment of a relationship between flightlessness and smaller optic lobes requires further study. The optic lobe is also relatively small in volant taxa such as owls, parrots and some songbirds [1], [22], [44].

Telencephalic volume has been positively correlated with sociality [20]. Although CT data do not allow for calculation of telencephallic volumes, scaling of the digital endocasts allows for the relative sizes of the telencephalon of different species to be evaluated in dorsal and lateral views. The telencephalon of the only two solitary nesting species, the Killdeer and the Marbled Murrelet, do not appear relatively smaller than other closely related taxa. Although the EQ of the Killdeer (0.646; Table 3) is the lowest among sampled non-alcid charadriiforms, the EQ of the Marbled Murrelet (0.731) approaches the average EQ value calculated for pan-alcids (0.758). Thus, EQ's for these taxa also do not support a correlation between sociality and brain size in charadriiforms.

Because the cerebellum is involved in motor control [35], [74], Iwaniuk et al. (2004) predicted that the relative size of the cerebellum of flightless birds might be smaller than that of volant relatives within a clade. Although assessment of cerebellum size based on digital endocasts is subjective because much of the cerebellum is obscured by the telencephalon, differences in the posterior aspect of the cerebellum can be identified. Given that aerially flightless pan-alcids have reduced the number of modes of locomotion from four (flying, walking, sea-surface paddling, wing-propelled diving) to three, it is in this context, striking that the posterior cerebella of Mancallinae sp. and the Great Auk are among the largest in relative size in the sample (Figs. 2, 3, 7, 8, 9, 10, 11) and do not support a reduction of cerebellum size in charadriiforms that have lost aerial flight. The width of the posterior cerebellum of *H. toliapicus* and all sampled alcids is greater than that of other sampled charadriiform taxa (16∶2). More generally in Aves, the posterior cerebella of flightless palaeognaths including rhea, moa, and ostrich are quite large in comparison with the telencephalon of those taxa [7], [13]. In contrast, the posterior aspect of the cerebella of penguins (e.g., *Pygoscelis antarctica*), the only extant flightless wing-propelled diving clade, are relatively small [17], [45]. The Common Murre *Uria aalge* and the Herring Gull also possess relatively larger posterior cerebella than other sampled charadriiform taxa (15∶1), although less posterodorsally expanded than in flightless charadriiforms (Figs. 3, 9). Therefore, current data do not support a smaller cerebellum in all flightless taxa, but instead, a more complex pattern within and among clades meriting further study.

An additional brain region that displayed considerable variation was the floccular lobe of the cerebellum (Fig. 13). Expanded floccular lobes have been functionally linked with the vestibulo-ocular (VOR) and vestibulocollic (VCR) reflexes [2], [75]. VOR and VCR are, in turn associated with agility of head and neck movements, gaze stabilization, and propioreception that facilitate the acrobatic maneuverability necessary for active pursuit of prey in many species. Differences between terrestrial foraging, aerially foraging and wing-propelled diving charadriiforms might be expected. However, correlation between a larger or more elongate flocculus size and feeding behavior or locomotor ecology, are not apparent in the charadriiform sample. For example, the floccular lobes are relatively elongate in wing-propelled diving Alcinae (except the Marbled Murrelet) and Mancallinae sp.; however they are also elongate in the Bridled Tern *Sterna anaethetus* and the Long-tailed Skua, as well as the enigmatic extinct species *H. toliapicus*. The floccular lobes are relatively shorter in another wing-propelled diving alcid subclade, Fraterculini, as well as the sampled gulls, terrestrial foragers, and the Black Skimmer (18∶2; Figs. 2, 3, 7, 8, 9, 10, 11, 13). Thus, flocculus size varied among wing-propelled divers (e.g., Marbled Murrelet vs. other Alcinae), among generalist or opportunistic feeders (e.g., Herring Gull vs. Long-tailed Skua), and between skimmers and plunge divers (e.g., Black Skimmer vs. Bridled Tern). For example, alcids are all characterized by rather poor aerial maneuverability [76], [77], but display a range of floccular morphology. However, species differences with respect to underwater acrobatics (e.g., variability and frequency of pitch, yaw, and roll maneuvers) that might explain differences in the flocculus in the clade have not been studied (though see [32], [33]). The high degree of variation in charadriiform floccular morphology suggests that factors other than locomotion and feeding strategy have influenced the anatomy of this structure.

### Orientation of Charadriiform Endocranial Regions

Variation in the relative position of brain regions was also identified. The telencephalon, optic lobe, and endosseous labyrinth are positioned in two distinct conformations in Charadriiformes. The main axis of the brain approached horizontal in most surveyed taxa (e.g., Razorbill Auk; Fig. 2); however, the main axis of the brains of some birds (e.g., cormorants; see [43]) are closer to horizontal. By contrast, the brain regions and endosseous labyrinth of the Tufted and Horned Puffins as well as several terrestrial foraging charadriiform species including the Killdeer, Australian Pratincole *Stiltia isabella* and the Painted Snipe *Rostratula benghalensis*, have a more vertically oriented main axis of the brain (24∶0; Figs. 2, 3, 7, 8, 9, 10, 11). The telencephalon of taxa with vertical brain orientation is less anteriorly expanded, the rhombencephalon extends along the entire ventral margin of optic lobe, and the endosseous labyrinth is positioned more anteriorly (i.e., ventral to the optic lobe). That *Fratercula* (puffins) alone among Pan-Alcidae show this vertical orientation is more striking given its absence in sampled Lari outgroup taxa to Pan-Alcidae (e.g., *Larus, Sterna, Rynchops, Stercorarius*). Puffins are behaviorally similar to other pan-alcids and the reason(s) for this difference in neural anatomy are unclear. If it were not for the possession of this characteristic by *Fratercula*, these differences might be interpreted as a reflection of differences in head orientation between more upright standing terrestrial foraging taxa and other charadriiforms that spend considerably more time in a more horizontally oriented position while on the wing or on the surface of the water. Further investigation of the anatomical differences between puffins and other pan-alcids and the potential reasons for those differences warrant additional study.

Variability in orientation of brain regions is extensive in other Aves but has not been discussed in detail, and its significance or potential relationship with an aspect of life history or ecology is unclear. The vertically oriented condition was previously noted in Scolopacidae [74], and therefore, based its presence in *Charadrius* and current hypotheses of charadriiform relationships [27], [28], it would be optimized as the ancestral condition for Charadriiformes. A vertical long axis orientation of the brain is also seen in the Rock Dove and the Common Myna *Acridotheres tristis* [43]. A horizontal long axis orientation of the brain is seen, for example, in the Great Horned Owl, New Caledonian Crow, Great Cormorant, and the Common Loon, but also the Mallard, Green Pheasant *Phasianus versicolor*, and the Red Jungle Fowl *Gallus gallus* [2], [23], [43]. The considerable homoplasy displayed by these characters suggests they are not phylogenetically distributed.

### Inner Ear Variation in Charadriiformes

The semicircular canals of sampled taxa varied in the number of common crus present (Fig. 4, 14, 15). Nineteen species of charadriiforms (Table 1) could be evaluated for this character and among those, all but three species possessed three common crus (i.e., crus between the horizontal, anterior, and posterior canals respectively; 26∶1). There was no junction between the anterior and the horizontal semicircular canals of the Pigeon Guillemot, Common Murre, and Black Skimmer. Based on the distribution of this character and because it has not been previously noted in Charadriiformes, the significance of having only two common crus is uncertain. Furthermore, the angle of the junction between the posterior semicircular canal and the anterior semicircular canal is more acute (∼35°) in Mancallinae sp. and the Horned Puffin than in other sampled taxa (∼50–80°; Figs. 4, 14, 15).

The acrobatic flight of taxa such as *Larus* and *Columba* has been hypothesized to be associated with relatively longer, and thinner semicircular canals (i.e., large overall circumference and smaller canal radius), whereas taxa with relatively shorter and broader semicircular canals such as *Anser*, *Anas*, and *Gallus* are characterized by more level, straight-line flight [8], [78], [79]. Even though extant alcids are also characterized by relatively fast, straight-line, non-acrobatic aerial flight [76], [77] the semicircular canals of alcids are quite long and thin like those of *Larus* and other sampled charadriiforms (Figs. 4, 14, 15). The similarity between the semicircular canals of wing-propelled diving and non-wing-propelled diving charadriiforms is similar to the reported absence of significant alterations of the vestibular system of penguins as compared to volant birds [19]. An outlier, Mancallinae sp. showed relatively shorter and more robust semicircular canals than other sampled charadriiforms (Figs. 4, 14, 15), and the systematic position of Mancallinae as the sister taxon to all other Pan-Alcidae suggests that this characteristic may be unique to that clade. Alternatively, it may be discovered to represent retention of an ancestral character state gained in the poorly sampled stem lineage of Alcidae early in the evolution of wing-propelled diving, then subsequently reversed.

The cross-sectional shape of the semicircular canals (25∶1) of Pan-Alcidae and extinct *H. toliapicus* are more compressed that of all other sampled charadriiforms (Figs. 4, 14, 15). Among taxa sampled, compressed semicircular canals is proposed to be apomorphic of Pan-Alcidae, thus potentially supporting inclusion of *H. toliapicus* in that clade. Indeed, Walsh and Milner (2011b) noted the compressed semicircular canals of *H. toliapicus* are unlike those of extant larids and that the relatively short semicircular canals (particularly the anterior semicircular canal) of *H. toliapicus* might be indicative of a relatively non-acrobatic, straight-line flyer. While the relatively large circumference and short length of the anterior semicircular canal of *H. toliapicus* is unlike that of any taxon sampled, the conclusion that *H. toliapicus* may have been a straight-line flyer [9] is congruent with the aerial flight of extant alcids [76], [77]. Compression of the semicircular canals seen in alcids may be generally related to increased sensitivity to head (and body) orientation related to efficient underwater pursuit of prey. It is also seen in diving ducks (e.g., *Aythya*) suggesting additional comparisons with other divers such as penguins, sulids, cormorants, and anseriforms are needed to further evaluate the systematic position of *H. toliapicus*.

The anterior semicircular canal of diving birds has been proposed to be dorsoventrally compressed and anteroposteriorly lengthened relative to terrestrial taxa, and the common crus is more deviated posteriorly [19]. In comparison with the relatively shorter anterior canals of terrestrial vertebrates, the increased length of the anterior canal may make it more sensitive to motion, suggesting that this adaptation is a response to the more complex ways that fully aquatic vertebrates rotate during underwater locomotion [19]. The results of previous research suggest that the height of the common crus and the height of the peak of the canal adjacent to the common crus are reduced in aquatic taxa relative to terrestrial taxa and that the presence of this morphological difference “in four broadly divergent groups of amniotes with terrestrial and secondarily aquatic members suggests that this represents some form of change that is an adaptation to the invasion of an aquatic environment” ([80]∶142). This proposed pattern is not observed in the charadriiform sample (Figs. 4, 14, 15). Although compressed semicircular canals are present only in Pan-Alcidae and *H. toliapicus* in this sample, the height and length of the anterior semicircular canals, as well as the position of the common crus of wing-propelled diving pan-alcids is proportional to that of other charadriiforms. Denser taxonomic sampling across Aves is needed to evaluate the distribution of endosseous labyrinth variation with respect to aquatic diving versus terrestrial foraging ecologies.

Additional differences in the endosseous labyrinths of surveyed taxa included the curvature of the cochlear duct and the shape of the distal tip of the cochlea (Figs. 4, 14, 15). The cochlear ducts of the Black Skimmer, Razorbill Auk, Great Auk, Common Murre, and Mancallinae sp. are relatively straight (27∶1) in comparison to the more curved cochlear ducts of other sampled charadriiforms. Curvature of the cochlear duct may reflect an elongation of the basilar papilla and an increase in the range of hearing frequency as has been shown in other birds [3], [81]. The distal end of the cochlear duct of both terrestrial charadriiforms (Australian Pratincole and Killdeer) is swollen (28∶1) in comparison with the more terete distal cochlear duct of other taxa sampled. No differences between the endosseous labyrinths of flightless and volant pan-alcids were noted; however, the cochlear duct of the Great Auk is relatively longer and more gracile than those of other sampled taxa.

Because endosseous labyrinth characteristics have been correlated with complexity of vocalization and sociality [3], differences in the hearing apparatus of gregarious, colony nesters and solitary nesters might be expected. However, conspicuous morphological differences between the endosseous labyrinths of solitary nesters (Marbled Murrelet, Killdeer, Great Horned Owl) and colonial nesters (Table 1) were not apparent in this sample. The morphological similarity of endosseous labyrinths and the lack of relative differences in EQ (see discussion above) between gregarious and more solitary charadriiforms suggests that the proposed link between sociality and endocranial morphology may not be universal among Aves (i.e., not present in charadriiforms); however, denser taxonomic sampling is needed to further evaluate that hypothesis.

### Black Skimmer Brain Morphology

The endocranial morphology of the Black Skimmer *Rynchops niger* is notable for its striking differences from other sampled charadriiforms. *R. niger* has a relatively larger wulst than any other sampled charadriiform (Figs. 2, 3, 7, 8, 9, 10, 11, 16). Although the encephalization quotient calculated for *R. niger* (EQ  = 0.708) is near the average for sampled charadriiforms (EQ  = 0.815), the wulst of the Black Skimmer approaches the large size of those in sampled outgroup taxa, *Bubo* and *Corvus*. Furthermore, the occipital sinus of the Black Skimmer is larger and more conspicuous than other sampled charadriiforms, and the endosseous labyrinth of the Black Skimmer does not contact the posterior margin of the optic lobe (Figs. 8, 16). In all other sampled taxa, including the outgroup taxon the Great Horned Owl, the anterior semicircular canal contacts the posterior margin of the optic lobe or is partially occluded by the optic lobe in lateral view. The optic lobe of the Black Skimmer is relatively smaller than that of other charadriiforms and similar in size to flightless pan-alcids. Remarkably, the endocranial differences between *R. niger* and other charadriiforms are far more pronounced than the differences between wing-propelled diving and other non-wing-propelled diving charadriiforms.

The Black Skimmer is known for unique attributes of the visual system as well as feeding ecology. Feeding is done in flight by skimming the surface of the water with an elongate and specialized lower jaw. It is the only avian taxon that is known to reduce its pupil to a vertical slit to reduce glare, and this tactile foraging species also engages in nocturnal feeding [26], [82], [83]. Pupil specialization, in conjunction with the small optic lobes, and occasional nocturnal feeding behavior of skimmers suggests that vision does not play a key role in the tactile feeding strategy of these birds. Telencephalic enlargement has also been documented in tactile-feeding scolopacid charadriiforms that are also know to forage in darkness (e.g., Dunlin *Calidris alpina*; [50]). However, the large anteriorly positioned wulst of *R. niger* contrasts with the relatively small and posteriorly positioned wulst of Scolopacidae such as the Dunlin and Eurasian Woodcock [44], [50]. Furthermore, a relatively enlarged wulst and smaller, or average sized optic lobes are known in select nocturnally foraging birds such as owls and some caprimulgiforms in which the wulst may play a role in the mediation of stereopsis [12]. The distribution of stereopsis in other clades is uncertain and the neural specializations of the Black Skimmer and whether these specializations are related to tactile feeding, the visual system (e.g., stereopsis), or shifts in proprioception merit further investigation.

## Conclusions

Despite the apparent plasticity of endocranial characteristics in response to behavioral attributes and recovered levels of homoplasy in endocranial characters, the position of the olfactory bulb, size of the optic tract, shape of the telencephalon, and width of the cerebellum in charadriiforms appear derived relative to compared outgroups. Within Charadriiformes, conspicuous differences in cerebellar morphology, the shape of contacts between regions of the brain (e.g., telencephalon and optic lobe), and the compression of the semicircular canals are evident between the endocranial anatomy of wing propelled diving Pan-Alcidae and other Charadriiformes (Fig. 16). Differences among the relative size of the brain, morphology of the wulst, and the relative size of the optic lobe of volant and flightless wing-propelled diving pan-alcids are also apparent. The degree of morphological disparity between volant and flightless pan-alcids is less striking than the differences between Pan-Alcidae and other Charadriiformes, which maybe due to continued utilization of the flight stroke in underwater propulsion. The combination of a large wulst, small optic lobe and conspicuous occipital sinus of the Black Skimmer is unlike the morphology of any other charadriiform sampled. Differences between the Black Skimmer and all other charadriiforms are more pronounced than those between wing-propelled diving Pan-Alcidae and other non-diving charadriiforms and likely related to the sensory demands of its unique feeding ecology.

In marked contrast to the brain, the morphology of charadriiform endosseous labyrinths is remarkably conserved among taxa with disparate ecologies (e.g., terrestrial foragers, generalists, wing-propelled divers, skimmers, plunge divers); the one exception being the compression of the semicircular canals noted in wing propelled diving Pan-Alcidae and the extinct species *H. toliapicus*. For example, the endosseous labyrinth morphology of the Black Skimmer is not markedly dissimilar from other charadriiforms, despite the derived morphology of the brain in this taxon. This pattern of variable brain morphology and conserved endosseous labyrinth morphology is also seen in penguins [17], [45]. While shifts in visual and propioreceptive stimuli appear to have profound effects on endocranial anatomy in these taxa, endosseous labyrinth morphology and likely sensory processing related to hearing and head orientation is generally conserved among wing-propelled diving forms and other Charadriiformes.

Relationships explored among endocranial anatomy, ecology, and ethology suggest that additional investigation of specializations in sensory systems and associated behaviors in birds may provide new insights into the evolution of these systems. Potential neuroethological correlations with attributes such as absolute genome size, sociality, wing-loading, and longevity should be be investigated. More detailed comparisons between sister taxa or closely related species that exhibit generalist and highly specialized behaviors may prove valuable for teasing apart the relative contributions of phylogeny and ecology with respect to the endocranial anatomy of birds and the path by which avian sensory systems have evolved.

## Supporting Information

Appendix S1 **Morphological Characters Identified from the Charadriiform Endocranium.**
(DOCX)

Appendix S2 **Endocranial Morphological Character Scorings.**
(DOCX)

Appendix S3 **Additional Details of Variation in the Charadriiform Endocranium.**
(DOCX)
