# Supplementary material for: Endocranial Anatomy of the Charadriiformes: Sensory System Variation and the Evolution of Wing-Propelled Diving
Source: PLoS One. 2012 Nov 27;7(11):e49584. doi: 10.1371/journal.pone.0049584 (PMC3507831; doi:10.1371/journal.pone.0049584)
Supplement: Appendix S1 — Morphological Characters Identified from the Charadriiform Endocranium. (DOCX) [file pone.0049584.s001.docx]

**Appendix S1. Morphological Characters Identified from the Charadriiform Endocranium**

1. Brain, olfactory bulb, size: (0) greatly reduced; (1) relatively enlarged. Although the reconstruction of olfactory bulbs for many sampled taxa were somewhat tentative due to a partial lack of bony architecture in that region of the skull, the size of the reconstructed olfactory bulbs of *Rissa tridactyla* and *Stiltia isabella* appear relatively smaller (i.e., olfactory bulbs protrude less anteriorly) than those of other sampled taxa (e.g., *Uria aalge*; Figs. 2, 3, 7-11, 15).

2. Brain, olfactory bulb, position: (0) ventral; (1) dorsal. The olfactory bulb of all sampled charadriiforms and *Halcyornis* *toliapicus* is positioned more dorsally than that of *Bubo virginianus* and *Corvus* *moneduloides*.

3. Brain, olfactory bulb, conformation: (0) clearly bifurcated; (1) single tract. Among sampled pan-alcids, only the olfactory bulb of *Uria aalge* is clearly bifurcated. This character displays considerable variation among sampled outgroup charadriiforms (i.e., non-pan-alcids).

4. Brain, telencephalon, shape in dorsal view: (0) rounded; (1) anteriorly tapered. In dorsal view, the telencephalic hemispheres of sampled charadriiforms are anteriorly tapered (i.e., broader posteriorly than anteriorly) in comparison with the more evenly rounded telencephalic hemispheres of *Bubo virginianus* and *Corvus* *moneduloides*.

5. Brain, wulst (sagittal eminence), dorsal expansion in anterior view: (0) not expanded; (1) distinctly enlarged. Among sampled Pan-Alcidae, the wulst of the murrelets *Brachyramphus marmoratus* and *Synthliboramphus antiquus* are less dorsally expanded than those of other pan-alcids, and the interhemispherical fissure of these taxa is somewhat shallow (i.e., the dorsoventral height of the wulst is less than the mediolateral width of the wulst). The wulst of *Sterna anaethetus*, *Stercorarius longicaudus*, *Stiltia isabella*, and *Charadrius vociferus* are also relatively non-distinct.

6. Brain, wulst, anteroposterior development in dorsal view: (0) extends from olfactory bulb to cerebellum; (1) positioned posteriorly; (2) positioned midway (does not contact olfactory bulb or cerebellum); (3) anteriorly positioned. The wulst of most sampled taxa extends anteroposteriorly from the olfactory bulb to the cerebellum. However, the wulst of *Synthliboramphus antiquus* and *Stercorarius longicaudus* is posteriorly positioned and the wulst of the only two sampled terrestrial taxa (*Stiltia isabella* and *Charadrius vociferus*) is positioned midway between the olfactory bulb and the cerebellum. The wulst of *Bubo virginianus* is positioned further anteriorly than in all sampled charadriiforms (see Iwaniuk and Wylie, 2006, figure 2).

7. Brain, wulst, lateral expansion of anterior wulst in dorsal view: (0) not expanded laterally; (1) expanded or swollen laterally beyond lateral margin of the telencephalon. The lateral margin of the anterior portion of the wulst (posterodorsal to the olfactory bulb) is laterally expanded past the lateral margin of the telencephalon in *P. impennis*, the closely related species *A. torda* (Smith and Clarke, 2011), Mancallinae sp., and *R. niger*.

8. Brain, junction between telencephalon and cerebellum in dorsal view: (0) v-shaped; (1) curved. Only the Alcinae display a curved contact between the telencephalon and cerebellum in dorsal view (contents of Alcinae include *Alca*, *Pinguinus*, *Alle*, *Uria*, *Cepphus*, *Brachyramphus*, and *Synthliboramphus*; Smith 2011b).

9. Brain, posterior telencephalon, shape in dorsal view: (0) rounded; (1) pointed. The posterior margin of the telencephalon of *Brachyramphus marmoratus* is more distinctly pointed than in any other sampled taxon. The osseous external morphology of the skull of brachyramphine murrelets does not reflect this soft-tissue endocranial feature and the significance of this feature is unclear at this time.

10. Brain, contact between telencephalon and dorsal optic lobe in lateral view: (0) straight; (1) curved. As noted by Walsh and Milner (2011b), the shape of the contact between the telencephalon and the optic lobe varies among Aves. Among taxa sampled, only the Pan-Alcidae, *Rynchops niger*, *Bubo virginianus*, and *Halcyornis* *toliapicus* display a curved contact between the telencephalon and the dorsal optic lobe.

11. Brain, relative size of optic lobe in lateral view (relative to rhombencephalon): (0) small; (1) large. The optic lobe is relatively smaller in the flightless pan-alcid taxa *Pinguinus impennis* and Mancallinae (i.e., the anteroposterior length of the optic lobe is less than the anteroposterior length of the rhombencephalon). *Rynchops niger* also has a relatively smaller optic lobe than in other sampled charadriiforms (e.g., *Larus argentatus*).

12. Brain, optic lobe in dorsal view: (0) visible; (1) not visible. The optic lobe is occluded from view by the telencephalon in dorsal view in all sampled species except *Charadrius vociferous*.

13. Brain, occipital sinus: (0) not visible; (1) clearly demarcated on dorsal cerebellum. Unlike the distinct occipital sinus that is visible along the mid-line of the cerebellum of many charadriiforms (e.g., *Rynchops niger*), no indication of the occipital sinus is visible on the virtual endocasts of *Stercorarius longicaudus* or sampled pan-alcids.

14. Brain, dorsal projection of cerebellum in lateral view: (0) >50% of posterior margin of telencephalon in contact with cerebellum; (1) <50% of posterior margin of telencephalon in contact with cerebellum. The dorsal extension of the cerebellum varies in the extent to which it contacts the posterior margin of the telencephalon. The cerebella of *Stiltia isabella*, *Charadrius vociferus*, and *Fratercula corniculata* are dorsally restricted compared to those of other sampled taxa (e.g., *Aethia cristatella*) in which more than half of the posterior telencephalon is in contact with the cerebellum (evaluated based on the dorsoventral height of the cerebellum with the brain oriented horizontally as indicated by the horizontal semicircular canal).

15. Brain, relative length of telencephalon and cerebellum in lateral view: (0) short cerebellum (i.e., anteroposterior length of cerebellum less than the half the anteroposterior length of the telencephalon); (1) long cerebellum (i.e., cerebellum posteriorly expanded; anteroposterior length of cerebellum more than half the anteroposterior length of telencephalon). The cerebella of *Pinguinus impennis*, *Uria aalge*, Mancallinae, and *Larus argentatus* are more posteriorly expanded than those of other sampled taxa.

16. Brain, cerebellum, width in dorsal view: (0) narrow; (1) moderately wide; (2) wide. Relative to the width of the telencephalon, the cerebellum of sampled alcids and *Halcyornis* *toliapicus* is noticeably wider than that of other sampled charadriiforms (i.e., the mediolateral width of the cerebellum in dorsal view is approximately equal to the mediolateral width of a cerebral hemisphere). The cerebellum of other charadriiforms (e.g., *Charadrius vociferus* and *Burhinus oedicnemus*; Figs. 2, 3, 7-11, 14, 15) is not as wide as that of pan-alcids (i.e., the mediolateral width of the cerebellum in dorsal view is less than the mediolateral width of a cerebral hemisphere). The cerebella of *Bubo virginianus* and *Corvus* *moneduloides* are conspicuously narrower than that of all sampled charadriiforms (i.e., the mediolateral width of the cerebellum in dorsal view is less than half of the mediolateral width of a cerebral hemisphere).

17. Brain, cerebellum, fissures: (0) distinct; (1) indistinct. In dorsal or lateral view, the cerebellum of non-alcid charadriiforms is characterized by distinct fissures. These fissures are not evident on the digital endocasts of any sampled Pan-Alcidae.

18. Brain, cerebellum, floccular lobes, mediolateral length: (0) reduced; (1) short; (2) elongate. The flocculus of some sampled taxa (e.g., *Alca torda*) is much more elongate (mediolateral length more than twice dorsoventral height) than that of other charadriiforms in which the flocculus is noticeably shorter (e.g., *Aethia* and *Fratercula*; mediolateral length less than twice dorsoventral height). The flocculus of the only two sampled terrestrial foraging charadriiforms (*Stiltia isabella* and *Charadrius vociferus*) is truncated (mediolateral length less than dorsoventral height) in comparison with that of the other sampled taxa.

19. Brain, cerebellum, floccular lobes, fenestration: (0) absent; (1) present. A foramen is present in the flocculus of *Alca torda*, *Stercorarius longicaudus*, and *Sterna anaethetus* (Figs. 2, 3, 7-11, 14, 15; see ventral view of *Alca torda*).

20. Brain, cerebellum, floccular lobes (includes the rostral and caudal floccular arteries and floccular sinus), shape in posterior view: (0) triangular with tapered lateral margin; (1) rectangular with thickened dorsal and ventral margins. A robust flocculus with rounded margins is characteristic of all Alcinae except *Brachyramphus*. This character is variable among the charadriiform outgroup to Pan-Alcidae.

21. Brain, rhombencephalon, sulcus: (0) conspicuous; (1) inconspicuous. An anteroposteriorly oriented sulcus is present along the ventral surface of the rhombencephalon in ventral view. This sulcus is distinct in many taxa (e.g., *Rissa tridactyla*). In virtual endocasts of the endocrania of the Pan-Alcidae and its sister taxon Stercorariidae (represented by *Stercorarius longicaudus*), this sulcus is less clearly defined.

22. Brain, pituitary gland (hypophysis), contact with optic tract: (0) separated; (1) contacting. The ventral margin of the optic tract contacts the dorsal margin of the pituitary gland in pan-alcids (e.g., *Alca torda*). These structures are separated in other sampled charadriiforms (e.g., *Rynchops niger*). Outgroup taxa *Bubo virginianus* and *Corvus moneduloides* do not possess the large optic tract typical of Charadriiformes. Additionally, the optic foramen of many charadriiforms is not completely ossified (Hall et al, 2009) and may have some correlation with contact between the optic tract and the pituitary body as reconstructed in these endocasts.

23. Carotid artery, entrance into pituitary gland fossa: (0) single; (1) paired. The conformation of the carotid arteries is known to vary among Aves and within Charadriiformes [85], and the reconstruction of the entrance of the ascending carotid artery into the pituitary gland fossa differs among sampled taxa. In *Bubo virginianus*, *Charadrius vociferus*, *Rynchops niger*, and *Rissa tridactyla*, the carotid is evident as a single artery (i.e., anastomosis of carotid rami ventral to pituitary gland); whereas in other sampled charadriiform species (e.g., *Alca torda*) paired carotid rami ascend into the pituitary gland fossa.

24. Brain and endosseous labyrinth, relative position and orientation: (0) vertical long-axis; (1) horizontal long-axis. The telencephalon of *Charadrius vociferus*, *Stiltia isabella*, and *Fratercula corniculata* are less anteriorly expanded in relation to the optic lobe and the endosseous labyrinths of these species are positioned more anteriorly. The majority of the endosseous labyrinth is positioned ventral to the optic lobe rather than posterior to it, in these species. Therefore the long-axis of the endocranium is approximately vertical (~75°-85° from horizonatal) in these three species. These regions of the endocranium are aligned more horizontally (20° from horizontal) in other sampled taxa (e.g., *Pinguinus impennis*), with the long-axis nearly horizontal in Mancallinae.

25. Endosseous labyrinth, semicircular canal, cross-sectional shape in lateral view: (0) rounded; (1) compressed. The semicircular canals of all sampled pan-alcids and *Halcyornis* *toliapicus* are more compressed than in other sampled charadriiforms (e.g., *Stercorarius longicaudus*). Thus it would appear that this character is an apomorphy of Pan-Alcidae relative to other Charadriiformes.

26. Endosseous labyrinth, semicircular canals, number of common crus: (0) two (asc-psc & hsc-psc); (1) three (asc-psc & hsc-psc & asc-hsc). Among sampled taxa, only *Cepphus columba*, *Uria aalge*, and *Rynchops niger* possess only two common crus, those between the anterior and posterior semicircular canal, and between the horizontal and posterior semicircular canals. All other sampled taxa (e.g., *Alca torda*) possess a third common crus between the anterior and horizontal semicircular canals.

27. Endosseous labyrinth, cochlear curvature in lateral view: (0) curved; (1) straight. Within Pan-Alcidae, a relatively straight cochlea is present in Mancallinae, and in three of the four Alcini species sampled (contents of Alcini include *Alca*, *Pinguinus*, *Alle*, *Miocepphus*, and *Uria*; Smith and Clarke, 2011). With the exception of *Rynchops niger*, the cochlea of all other sampled outgroup taxa to Pan-Alcidae were more curved.

28. Endosseous labyrinth, shape of distal cochlea in lateral view: (0) tapered; (1) swollen. Although less pronounced in *Stiltia isabella* than in *Charadrius vociferus*, a swollen distal tip of the cochlea is present only in these two terrestrially foraging charadriiforms among taxa sampled herein.
