# Supplementary material for: Endocranial Anatomy of the Charadriiformes: Sensory System Variation and the Evolution of Wing-Propelled Diving
Source: PLoS One. 2012 Nov 27;7(11):e49584. doi: 10.1371/journal.pone.0049584 (PMC3507831; doi:10.1371/journal.pone.0049584)
Supplement: Appendix S2 — Endocranial Morphological Character Scorings. (DOCX) [file pone.0049584.s002.docx]

**Appendix S2. Endocranial Morphological Character Scorings**

*Stiltia isabella* and *Charadrius vociferus* were not scored for character 20 because the flocculus of those species is so reduced in size that it is not applicable. *Corvus moneduloides* was not scored for characters 25-28 because those data are not currently available (i.e., no endocasts have been published for that species). *Bubo virginianus* and *Corvus moneduloides* were not scored for character 22 because of the reduced size of the optic tract in those taxa.

1 5 10 15 20 25

. . . . . .

*Alca torda* 1111101101110102121111111110

*Pinguinus impennis* 1111101101010112120111111110

*Alle alle* 1111100101110102120111111100

*Uria aalge* 1101100101110112120111111010

*Cepphus columba* 1111100101110102120111111000

*Brachyramphus marmoratus* 1111000111110112110011111100

*Synthliboramphus antiquus* 1111010101110102120111111100

*Aethia cristatella* 1111100001110102110011111100

*Fratercula corniculata* 1111100001110002110011101100

Mancallinae sp. 1111101001010112120111?11110

*Stercorarius longicaudus* 1111010000111101021110110100

*Sterna anaethetus* 1111100000111101021100110100

*Rynchops niger* 1101101001011101010100010010

*Rissa tridactyla* 0101100000111101010100010100

*Larus argentatus* 1111100000111111010000110100

*Stiltia isabella* 0101020000111001000-00100100

*Charadrius vociferus* 1111020000101001000-00000101

*Halcyornis toliapicus* 1101100001111102020110111101

*Bubo virginianus* 100013000101110011000-010101

*Corvus moneduloides* 001011000101000001000-11????
