# Supplementary material for: Endocranial Anatomy of the Charadriiformes: Sensory System Variation and the Evolution of Wing-Propelled Diving
Source: PLoS One. 2012 Nov 27;7(11):e49584. doi: 10.1371/journal.pone.0049584 (PMC3507831; doi:10.1371/journal.pone.0049584)
Supplement: Appendix S3 — Additional Details of Variation in the Charadriiform Endocranium. (DOCX) [file pone.0049584.s003.docx]

**Appendix S3. Additional Details of Variation in the Charadriiform Endocranium**

The posterior margin of the telencephalon is rounded in dorsal view in all sampled taxa except the marbled Murrelet *Brachyramphus marmoratus*, in which the posterior margin of the telencephalon is distinctly pointed (9:1; Figs. 2, 3, 7-11, 14, 15). Furthermore, the contact between the dorsal cerebellum and the midline of the posterior margin of the telencephalon is curved (8:1) or a ‘V’ shape in dorsal view. The contact between the telencephalon and the optic lobe may be curved (10:1; e.g., *Uria aalge*, Fig. 8) or straight (e.g., *Larus argentatus*, Fig. 8) in lateral view. The telencephalon extended laterally and occluded the optic lobe from view dorsally (11:0) in all sampled taxa except the Killdeer *Charadrius vociferous* (Fig. 6).

The wulst displayed considerable variation across the taxonomic sample and particularly within Pan-Alcidae (Figs. 2, 3, 7-11, 15). The degree to which the wulst projects dorsally in anterior view was conspicuously smaller (relatively) in some Pan-Alcidae (e.g., *Brachyramphus marmoratus*; 5:0), whereas in *Rynchops niger* the wulst was prominent, resulting in a rather deep interhemispherical fissure. The wulst also varied in its extent of anteroposterior prominence. Although the wulst of many charadriiform taxa extended along the entire anteroposterior length of the telencephalon (e.g., *Alca torda*; 6:0), the wulst of other taxa were positioned posteriorly (e.g., *Synthliboramphus antiquus*; 6:1), positioned midway along the telencephalon (e.g., *Stiltia isabella*; 6:2; i.e., restricted anteriorly and posteriorly), or anteriorly positioned (e.g., *Bubo virginianus*; 6:3). Additionally, the wulst of *Alca torda*, its flightless sister taxon *Pinguinus impennis*, the flightless alcid Mancallinae sp., and *Rynchops niger* extends laterally, past the lateral margin of the telencephalon (7:1).

The relative size and position of the optic lobe varies across Aves and among the charadriiform taxa sampled herein. The optic lobes of charadriiforms are relatively smaller than those of the tinamou [13], but are not completely covered in lateral view by the telencephalon as in *Apteryx* (kiwis) and *Dinornis* (moas; [18]). The width of the apparent contact between the optic lobe and the cerebellum is related to the occlusion of the optic lobe by the posterior telencephalon. In lateral view the optic lobe appears to posteriorly taper to a point in most charadriiform taxa (e.g., *Rissa tridactyla*), whereas in a few taxa (e.g., *Stiltia isabella*; Fig. 8) the contact between the posterior margin of the optic lobe and the cerebellum is broader. Flightless taxa *Pinguinus impennis* and Mancallinae sp. have relatively smaller optic lobes than other pan-alcids (12:0; Figs. 3, 9).

Pronounced variation was documented in the cerebellum of sampled charadriiform taxa; cerebellar morphology is known to be highly variable in Aves [34], [45], [46]. The occipital sinus is conspicuous on the dorsal cerebellum in some taxa (e.g., *Rynchops niger*; 13:1), whereas this feature was not discernable in other species (e.g., *Alca torda*). Likewise, cerebellar fissures were evident along the dorsal and posterior cerebella (17:0) of the sampled outgroup taxa to Pan-Alcidae but were not evident in pan-alcids. There is substantial variation in the relative maximum width of the cerebellum (in dorsal view), as compared to the telencephalon (Figs. 2, 3, 7-11, 14, 15). Additionally, the dorsal portion of the cerebellum varies in the extent that it contacts the posterior margin of the telencephalon. The cerebellum of *Stiltia isabella*, *Charadrius vociferus*, and *Fratercula corniculata* are dorsally restricted in comparison to those of other sampled taxa (e.g., *Aethia cristatella*), in which more than half of the posterior telencephalon is in contact with the cerebellum (14:0). All sampled charadriiform taxa displayed a distinct sulcus for passage of the semicircular vein (Figs. 2, 3, 7-11, 15). The length of the cerebellum relative to the telencephalon varied; in the majority of charadriiform taxa (e.g., *Alle alle*) the length of the cerebellum was less than 50% that of the telencephalon (15:0).

The floccular lobes of the cerebellum varied in their length, overall shape, and possession of a fenestra. Their lobes varied from rather small, irregularly triangular lobes with tapered edges (20:0), to relatively larger, more robust, approximately rectangular lobes with thickened dorsal and ventral margins (20:1). The floccular lobes of the two terrestrial charadriiform taxa sampled (*Stiltia isabella*, *Charadrius vociferus*) were noticeably smaller than those of other charadriiforms (mediolateral length less than dorsoventral height; 18:0; Fig. 14). Other terrestrial palaeognath taxa such as the tinamous, kiwis, and moas also lack well-developed floccular lobes [18]. Relatively long floccular lobes were evident in many pan-alcids (e.g., Mancallinae sp.) and *Halcyornis* *toliapicus* (mediolateral length more than twice dorsoventral height; 18:2). Large floccular lobes are also seen in some galloanserines that inhabit both terrestrial and aquatic niches (e.g., *Chauna chauvaria*, *Anas platyrynchos*, *Gallus gallus*, *Phasianus versicolor*; [18], [40]). Curiously, this feature is highly variable in penguins, with the floccular lobes of *Paraptenodytes antarcticus* and *Spheniscus humboldti* substantially larger than those of *Aptenodytes patagonicus* and *Pygoscelis antarctica* [17]. The flocculus of only three sampled species, *Alca torda*, *Sterna anaethetus*, and *Stercorarius longicaudus*, was fenestrated (19:1), the significance of which is unknown because this feature is reported here for the first time and its distribution within Aves is uncertain.

The olfactory bulb was variably bifurcated (3:0) or a single tract within Pan-Alcidae and its sister taxon, Stercorariidae (Figs. 2, 3, 7-11, 15). The olfactory bulbs of *Rissa tridactyla* and *Stiltia isabella* are relatively smaller than those of other sampled taxa (1:0; see Appendix 1 for quantification of relative size terms used throughout). The relative size range of charadriiform olfactory bulbs are comparable to that represented by *Chauna chavaria* and *Anas platyrhychos* within Anseriformes (relatively large to relatively small, respectively). The hypertrophied condition in *Apteryx mantelli* or *Struthio camelus* [13], [18] was not observed in this sample*.* Because the relative size of olfactory bulbs in birds is correlated more strongly with olfactory capabilities than with relative endocranial volume or body size [14], the range of olfactory bulb relative size in Charadriiformes suggests that there is variation in the degree to which different species in this sample depend on olfactory sense.

Additional morphological variation documented in sampled taxa included differences in the rhombencephalon, pituitary gland, and the carotid arteries. An anteroposteriorly oriented sulcus was evident along the ventral surface of the rhombencephalon many taxa (21:0; sulcus absent or indistinct in Pan-Alcidae, *Stercorarius*, and *Halcyornis toliapicus*). The paired carotid rami alternatively entered the pituitary gland (i.e., hypophysis) separately (23:1), or else they anastomosed prior to entry into the pituitary gland (23:0). Additional structures such as the paired glosopharyngeal and vagus nerves (IX-XI) and trigeminal nerve (V_2,3_) were discernable in the digital endocranial endocasts (Figs. 2, 3, 7-11, 14, 15). However, the relative size, position, and conformation of cranial nerves are relatively invariant among the sampled taxa. The relative size and position of the foramen magnum was also largely conserved among sampled taxa. Whereas the ventral margin of the optic tract and the dorsal margin of the pituitary gland of pan-alcids are in contact (22:1), these structures are separated in other charadriiforms.

In addition to morphological variation of individual brain regions, variation in the relative positions of brain regions was also identified. The relative positions of the telencephalon, optic lobe, and endosseous labyrinth conform to two distinct groups. The brain regions and endosseous labyrinth of several terrestrial charadriiform species *Charadrius vociferus*, *Stiltia isabella* and *Rostratula benghalensis*, and those in the wing-propelled diving alcids *Fratercula cirrhata* and *Fratercula corniculata*, are more vertically oriented or “stacked” than those of other sampled taxa (24:0; Figs. 2, 3, 7-11, 14, 15); the telencephalon of species these taxa is less anteriorly expanded, and the endosseous labyrinths are positioned more anteriorly (i.e., ventral to the optic lobe). By contrast, the telencephalon is positioned more anteriorly and endosseous labyrinths, largely posterior to the optic lobe in *Tringa stagnatilis* and *Larus crassirostris* (see Kawabe et al., 2009).

Morphological variation of the endosseous labyrinths of sampled charadriiform taxa was limited, with only four variable morphological characters identified (Appendices 1 & 2). The path of the anterior (rostral) semicircular canal is longer than that of the horizontal and posterior (caudal) semicircular canals in all surveyed taxa (Figs. 4, 12, 16), and the anterior semicircular canal of all sampled charadriiforms extends dorsally from the anterior ampulla and the crus with the posterior semicircular canal. There is an additional crus between the anterior semicircular canal and the horizontal semicircular canal distal to the origin of the anterior canal in some taxa, and the rostral and horizontal ampullae are distinctly swollen and separated by a narrow crevice.
